# Causal effects of circulating glutamine on colitis, IBD, and digestive system cancers: a Mendelian randomisation study

**DOI:** 10.7150/jca.96085

**Published:** 2024-05-20

**Authors:** Yang Xie, Yonghui Wu, Qing Tao, Youxiang Chen, Chunyan Zeng

**Affiliations:** 1 Department of Gastroenterology, digestive disease Hospital, The First Affiliated Hospital, Jiangxi Medical College, Nanchang University, Nanchang China.; 2 Jiangxi Clinical Research Center for Gastroenterology, Nanchang, Jiangxi, China.

**Keywords:** circulating glutamine, IBD, Mendelian randomization, Colitis, Digestive tumors, GWAS

## Abstract

**Backgrounds:** There is growing evidence linking glutamine levels to the risk of gastrointestinal diseases, yet the presence of a causal relationship remains uncertain. In this study, we employed a Mendelian randomization (MR) approach to investigate potential causal associations between glutamine and colitis, inflammatory bowel disease (IBD), and digestive tumors.

**Methods:** Genetic instrumental variables for glutamine exposure were identified from a genome-wide association study (GWAS) involving 114,751 participants. We pooled statistics from GWAS of gastrointestinal diseases in European populations, encompassing colitis (cases=1193, controls=461,740), IBD (cases=31,665, controls=33,977), Crohn's disease (cases=17,897, controls=33,977), ulcerative colitis (cases=1,239, controls=990), oesophageal cancer (cases=740, controls=372,016), gastric cancer (cases=6,563, controls=195,745), liver cell carcinoma (cases=168, controls=372,016), hepatic bile duct cancer (cases=418, controls=159,201), pancreatic cancer (cases=1,196, controls=475,049), and colon cancer (cases=1,494, controls=461,439). To ensure the validity of our findings, we utilized several analytical approaches including inverse variance weighted, weighted median, weighted mode, MR-Egger, and simple mode method.

**Results:** Using the IVW method, we found that glutamine levels were inversely associated with colon cancer (OR = 0.998; 95% CI: 0.997-1.000; P = 0.027), colitis (OR = 0.998; 95% CI: 0.997-1.000; P = 0.020), and IBD (OR = 0.551; 95% CI: 0.343-0.886; P = 0.014). Subgroup analysis revealed a negative association between glutamine and Crohn's disease (OR = 0.375; 95% CI: 0.253-0.557; P = 1.11E-06), but not with ulcerative colitis (OR = 0.508; 95% CI: 0.163-1.586; P = 0.244). Glutamine levels showed no significant correlation with oesophageal cancer (OR = 1.000; 95% CI: 0.999-1.001; P = 0.566), gastric cancer (OR = 0.966; 95% CI: 0.832-1.121; P = 0.648), liver cell carcinoma (OR = 1.000; 95% CI: 0.999-1.000; P = 0.397), hepatic bile duct cancer (OR = 0.819; 95% CI: 0.499-1.344; P = 0.430), and pancreatic cancer (OR = 1.130; 95% CI: 0.897-1.423; P = 0.301). Sensitivity analyses also supports this finding, affirming the reliability and robustness of our study.

**Conclusions:** This study suggests that blood glutamine levels in European populations may lower the risk of colon cancer, colitis, and IBD, particularly Crohn's disease. Nevertheless, additional research involving a diverse range of ancestries is imperative to corroborate this causal relationship.

## Introduction

Glutamine, a prominent amino acid in human plasma, plays crucial roles in maintaining human health by regulating energy metabolism, preserving acid-base equilibrium, and ensuring cellular integrity 1-3. It can undergo hydrolysis into glutamate and ammonium ions (NH4^+^) via glutaminase, while glutamate and ammonia (NH3) can be enzymatically converted back into glutamine by glutaminase 3. Research indicates a close association between glutamine and intestinal disorders 4. Studies have consistently shown lower serum glutamine levels in colorectal cancer (CRC) patients compared to healthy individuals 5-8. Furthermore, clinical investigations have linked serum glutamine deficiency to increased recurrence and metastasis of colorectal cancer 9, 10. A meta-analysis revealed that glutamine supplementation significantly enhances humoral and T-cell immune function indices in post-surgery CRC patients 11, potentially reducing complications and improving treatment outcomes 12. Furthermore, glutamine has been shown to alleviate symptoms of ulcerative colitis and Crohn's disease 13, 14. Glutamine analogues hold promise in the treatment of pancreatic cancer 15. However, some studies have reported increased side effects associated with glutamine supplementation 16. Prior research has yielded conflicting or inconclusive evidence regarding the correlation between glutamine and digestive disorders. This underscores the necessity for more comprehensive analyses to systematically evaluate the link between glutamine and the onset of intestinal diseases and cancer. Moreover, observational studies are prone to unmeasurable confounding and reverse causality, further obscuring the potential relationship between circulating glutamine levels and digestive diseases.

Mendelian randomization (MR) is an epidemiological method that infers potential causality by using genetic variants as instrumental variables 17. MR studies are less susceptible to confounding and reverse causation because genetic variants are randomly assigned at conception and remain unchanged thereafter 18. Although previous MR studies have found glutamine to be associated with the risk of thyroid cancer 19, its association with other gastrointestinal disorders, such as IBD, colitis, and gastrointestinal (GI) neoplasms, has yet to be determined. In this study, we aimed to explore the potential causal relationship between glutamine and colitis, IBD, and six major GI tumors through a two-sample MR analysis using genome-wide association study (GWAS) data.

## Materials and Methods

### Study design

**Figure 1:**
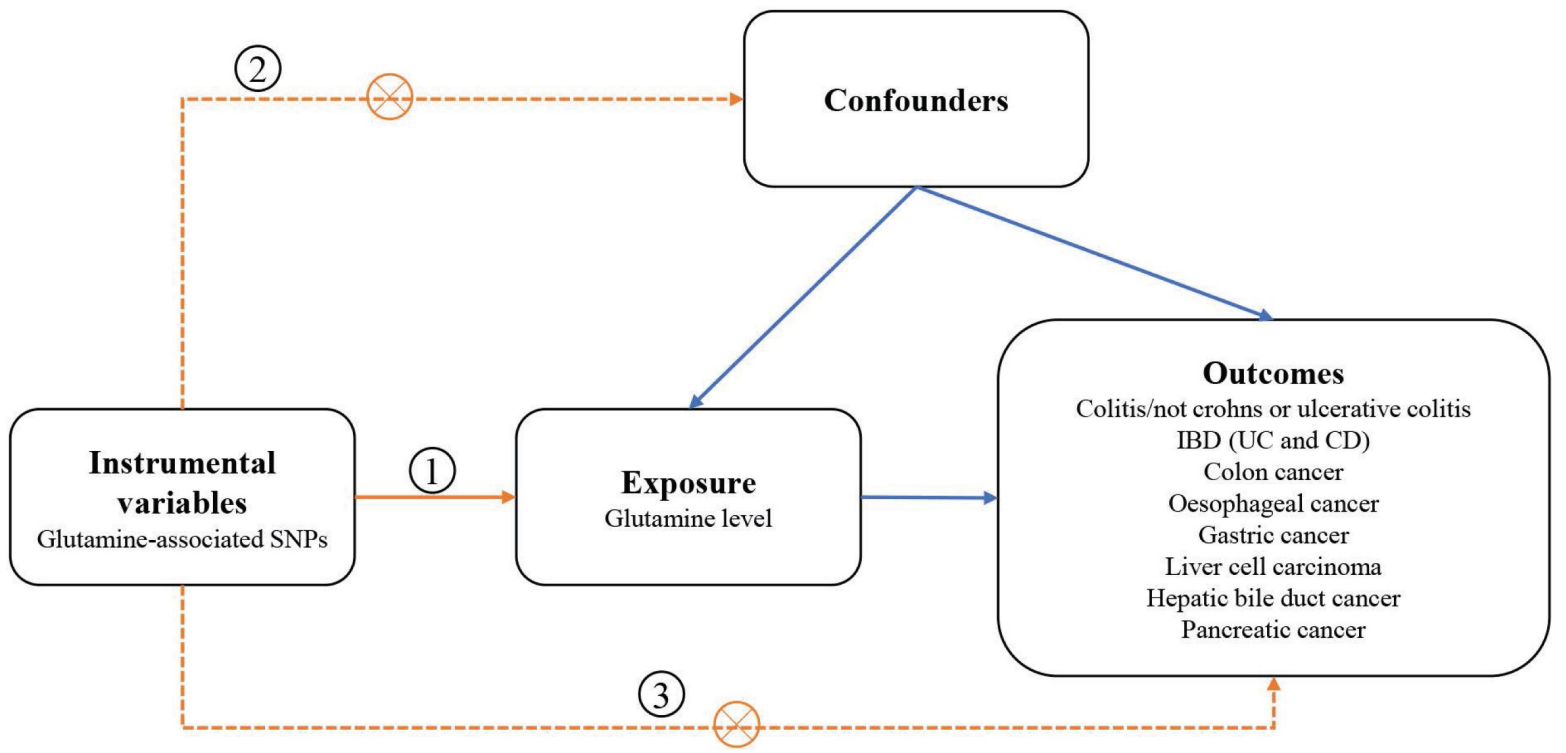
Schematic overview of the study design. SNPs single nucleotide polymorphisms; IBD inflammatory bowel disease; CD Crohn's disease; UC ulcerative colitis.

We designed a two-sample MR study to assess the causal impact of glutamine on digestive disorders (Figure 1). The MR design was subject to the following 3 assumptions:(1) genetic variation used as a genetic instrumental variable is strongly associated with glutamine; (2) genetic variation is independent of any confounders; and (3) genetic variation is associated with outcomes only through glutamine and not through any other causal pathway. Our data are largely based on independent GWAS.

### Genetic instrumental variables

The glutamine -associated genetic variants used in our study were derived from a GWAS analysis consisting of up to 114751 mixed populations containing 11590399 SNPs 20. These single nucleotide polymorphisms were at the genome-wide significance level (p<5×10^ -8^). In addition, among SNP pairs with linkage disequilibrium (LD), only SNPs with the lowest p-values were retained using an R^2^ threshold <0.05 (LD window of 5000 kb) 21. Additionally, we excluded palindromic SNPs with intermediate allele frequencies and calculated the F parameter to evaluate the strength of the instrument. SNPs with F values less than 10 were discarded due to their low statistical efficacy. Finally, 52 strictly selected SNPs were retained as genetic instrumental variables for causal analyses across all participants (Supplementary Table 1).

### Outcome data sources

We excluded cancer outcome data that overlapped with the exposed population to mitigate potential bias caused by overlap. Ultimately, our study encompassed six common types of digestive system cancer. Genetic data for gastrointestinal diseases were obtained from the Open GWAS website (https://gwas.mrcieu.ac.uk/datasets/), which includes the UK Biobank study 22. The UK Biobank is a large population-based cohort study including over 500,000 people 22. GWAS summary statistics for colon cancer comprised 1,494 cases and 461,439 controls of European origin (ukb-b-20145). Genetic summary statistics for colitis included 1,193 cases and 461,740 controls of European origin (ukb-b-3044). Genetic summary statistics for IBD included 31,665 cases and 33,977 controls of European origin 23. Genetic summary statistics for ulcerative colitis comprised 1,239 cases and 990 controls of European origin 23. Genetic summary statistics for Crohn's disease included 17,897 cases and 33,977 controls of European origin 23. Genetic summary statistics for oesophageal cancer comprised 740 cases and 372,016 controls of European origin (ieu-b-4960). Genetic summary statistics for gastric cancer comprised 6,563 cases and 195,745 controls (bbj-a-119). Genetic summary statistics for liver cell carcinoma comprised 168 cases and 372,016 controls (ieu-b-4953). Genetic summary statistics for hepatic bile duct cancer included 418 cases and 159,201 controls 24. Genetic summary statistics for pancreatic cancer comprised 1,196 cases and 475,049 controls 24. All digestive diseases outcomes were defined using International Classification of Diseases, Ninth (ICD-9) and Tenth (ICD-10) editions codes 25.

### Statistical analysis

We used several methods to estimate the potential causal relationship between glutamine and gastrointestinal disorders, including fixed/random-effects inverse variance weighted (IVW) methods, weighted median methods, MR-Egger regression, and the MR multiple-effects residual sum and outliers (MR-EMO) test. We used the IVW method as the main analysis because it provides the most accurate effect estimates and is used as the main analysis in almost all MR analyses 26-28. The IVW method first calculates ratio estimates for individual SNPs by using the Wald estimator and the Delta method, and then combines the estimates calculated from each SNP to obtain the main causal estimate 29. Heterogeneity between our selected SNPs was tested using Cochran's Q test, and if heterogeneity existed (p<0.05), the random-effects IVW method was selected, otherwise the fixed-effects IVW method was used 30. Since the results of IVW methods are susceptible to validated instruments and potential pleiotropic effects, we performed sensitivity analyses to assess the robustness of the correlations. First, we used MR-Egger regression to test for potential horizontal pleiotropy; if the p-value of the intercept is less than 0.05, horizontal pleiotropy of SNPs may exist 31. Then, we performed MR-STO test which performs a global test of heterogeneity to determine if there are possible outliers in the SNPs and to obtain corrected association results after removing potential outliers 32. To further assess the impact of potential directional pleiotropy, we used the GWAS catalogue (https://gwas.mrcieu.ac.uk/datasets/, last accessed on 2 March 2024) and performed MR analyses again after exclusion of SNPs associated with other phenotypes. Associations between glutamine and gastrointestinal disorders were expressed as a ratio of ratios (OR) and its 95% confidence interval (CI). All MR analyses were performed using R version 4.3.0 (https: //www.rproje ct.org/) with “Mendelian Randomization”, “TwoSampleMR” and “MR-PRESSO” software packages.

## Results

### Selection of instrumental variables

We extracted 52 SNPs as instrumental variables (IVs) from the glutamine dataset (ebi-a-GCST90092818) with a significance level of p < 5 × 10^-8^. Additionally, we calculated the F-statistic for each SNP, ranging from 29.83 to 2201.75, all surpassing 10, indicating robustness and alignment with our initial hypothesis (Supplementary Table 1). Detailed information on the SNPs associated with gastrointestinal diseases for the selected IVs, including p-values, β-coefficients, standard errors (SEs), and effector alleles, is provided in Supplementary Table 1. Lastly, for various outcome events—colon cancer, IBD, colitis, ulcerative colitis, Crohn's disease, oesophageal cancer, gastric cancer, liver cell carcinoma, hepatic bile duct cancer, and pancreatic cancer—we selected 19/3/15/5/3/37/30/28/32/41 SNPs as genetic instruments for MR analysis. The information regarding the glutamine-related gene variants and their effects on IBD, CD, colitis and colon cancer can be found in Tables 1, 2, 3, 4. The information concerning glutamine-related gene variants and their effects on ulcerative colitis, oesophageal cancer, gastric cancer, liver cell carcinoma, hepatic bile duct cancer, and pancreatic cancer included in the study can be found in Supplementary Tables 2, 3, 4, 5, 6, 7.

### The effect of glutamine levels on colitis, IBD, and digestive system diseases

**Figure 2:**
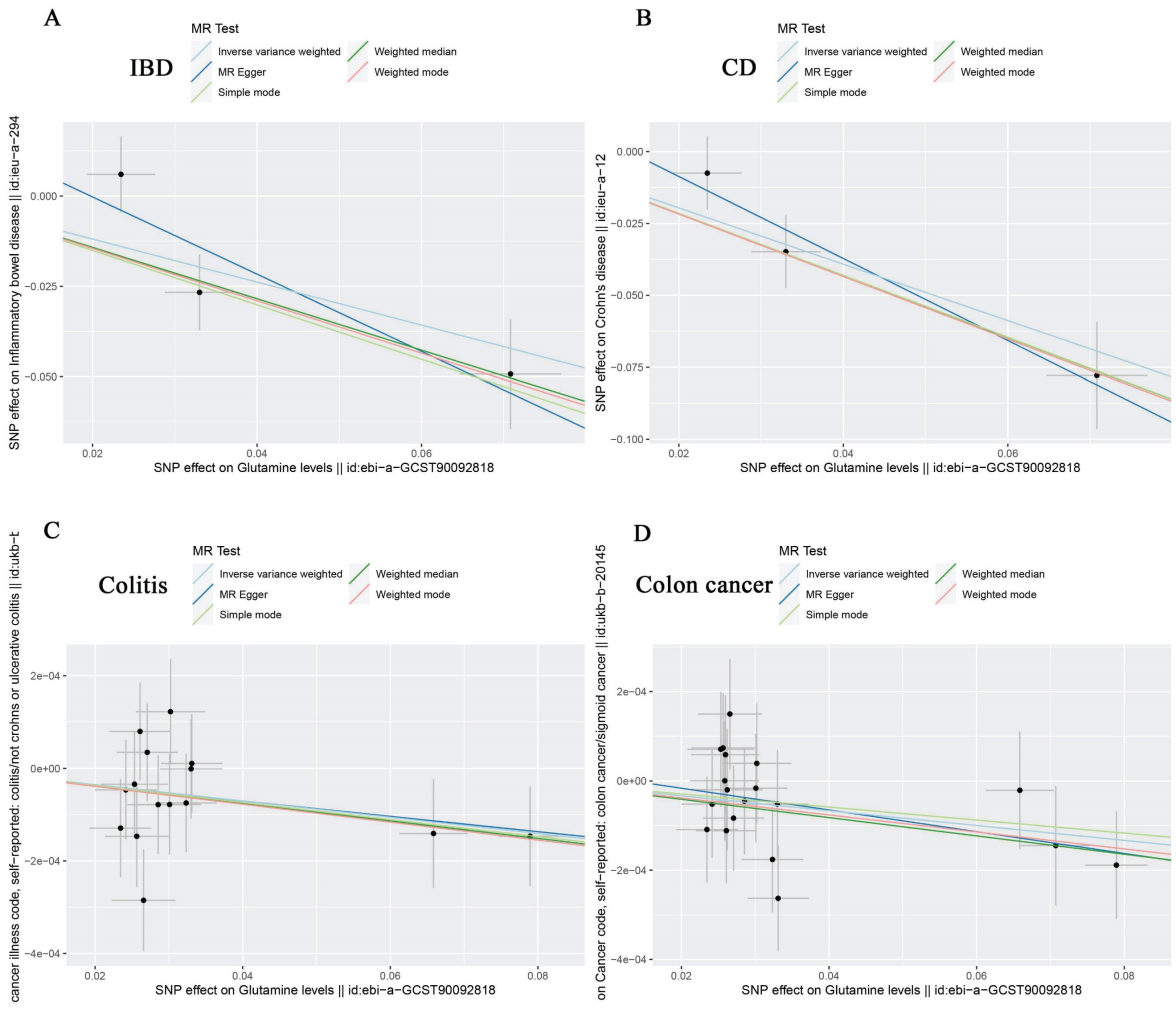
Scatter plots showing the causal effect of SNPs on glutamine (ebi-a-GCST90092818) against the effects on IBD (A), CD (B), Colitis (C) and Colon cancer (D). SNP single nucleotide polymorphisms; MR Mendelian randomization

**Figure 3:**
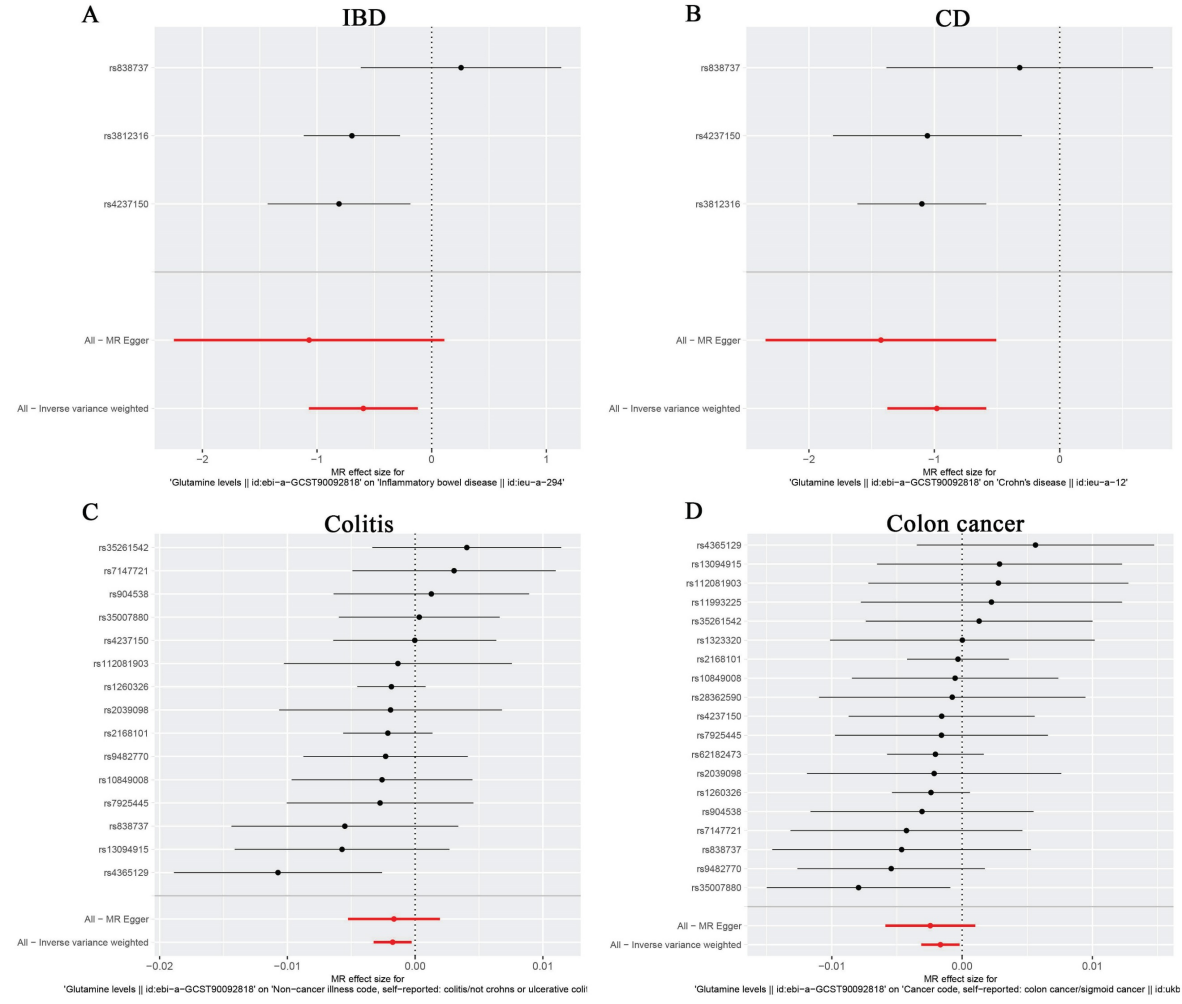
Forest plots of MR analyses from glutamine to IBD (A), CD (B), Colitis (C) and Colon cancer (D). The red points showed the combined causal estimate using all SNPs together in a single instrument, using two different methods (MR-Egger and IVW).

According to IVW analysis, there was no significant causal effect between glutamine levels and the genetic susceptibility to oesophageal cancer (OR = 1.000; 95% CI: 0.999-1.001; P = 0.566), gastric cancer (OR = 0.966; 95% CI: 0.832-1.121; P = 0.648), liver cell carcinoma (OR = 1.000; 95% CI: 0.999-1.000; P = 0.397), hepatic bile duct cancer (OR = 0.819; 95% CI: 0.499-1.344; P = 0.430), and pancreatic cancer (OR = 1.130; 95% CI: 0.897-1.423; P = 0.301) in the European population (Table 5). However, in subsequent analyses, we observed a negative correlation between glutamine levels and IBD (OR = 0.551; 95% CI: 0.343-0.886; P = 0.014), CD (OR = 0.375; 95% CI: 0.253-0.557; P = 1.11E-06), colon cancer (OR = 0.998; 95% CI: 0.997-1.000; P = 0.027), and colitis (OR = 0.998; 95% CI: 0.997-1.000; P = 0.020), with no significant correlation with UC (OR = 0.508; 95% CI: 0.163-1.586; P = 0.244) (Table 5). The scatter plots and forest plots were shown in Figure 2A-D, Figure 3A-D, Supplementary Figure 1, and Supplementary Figure 2. This suggests that glutamine may act as a protective factor against colon cancer, colitis, IBD, and especially CD in European populations. The results obtained by the weighted median approach for IBD and CD support these findings.

### Sensitivity analysis

**Figure 4:**
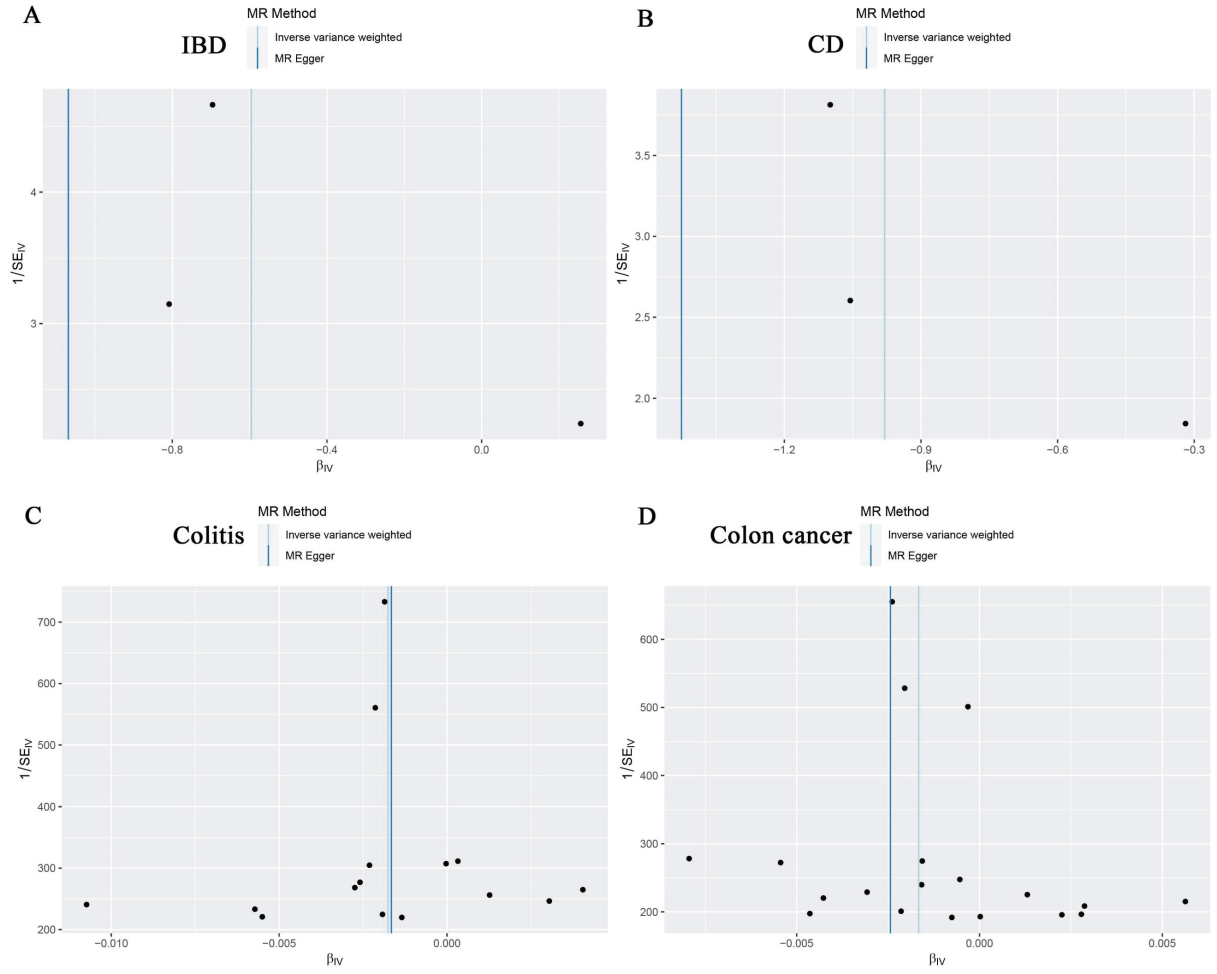
Funnel plots showing no significant heterogeneity among the SNPs of IBD(A), CD(B), Colitis(C) and Colon cancer(D). SE standard error

**Figure 5:**
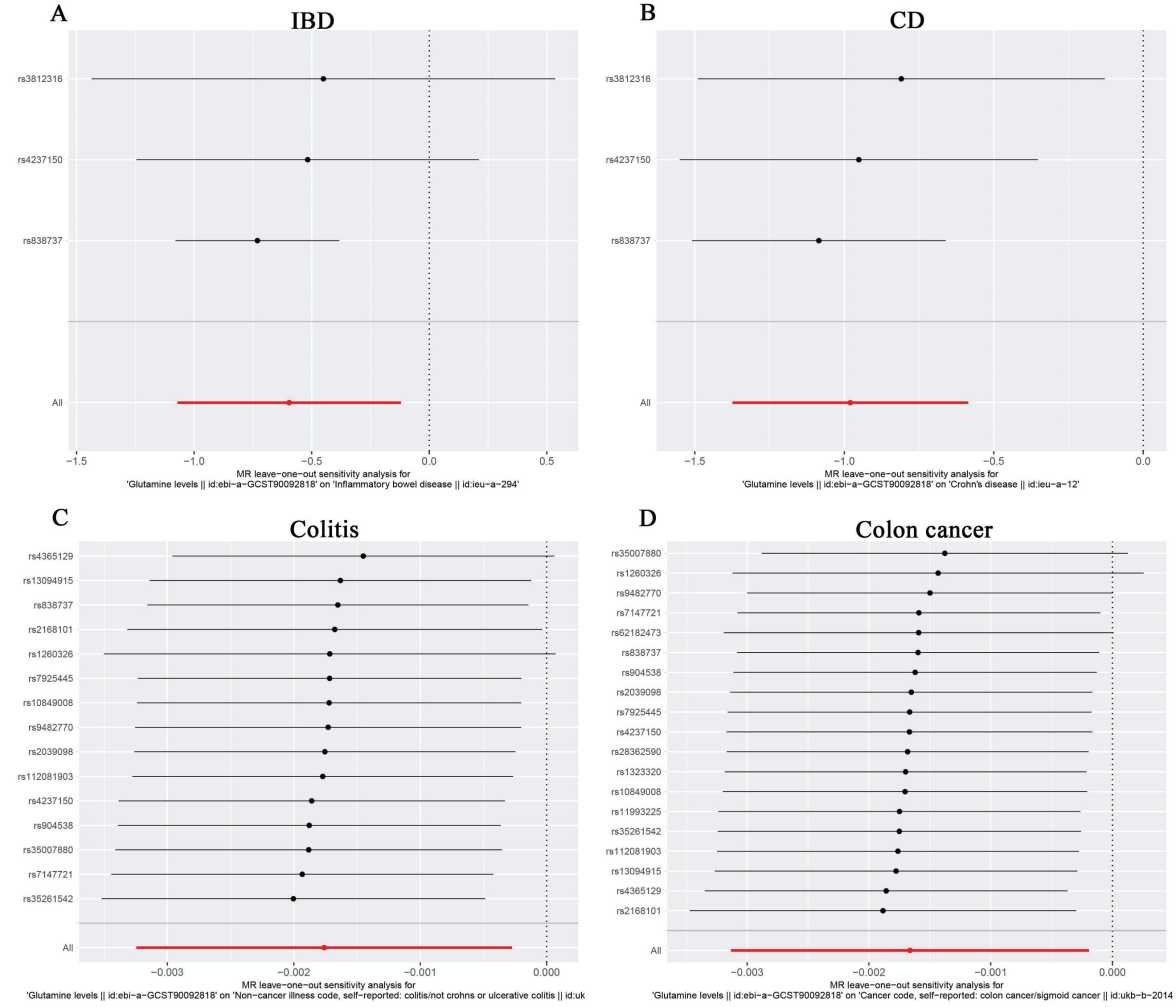
The Forest plot of leave-one-out sensitivity analysis showing the impact of each SNP on the overall causal estimate to IBD(A), CD(B), Colitis(C) and Colon cancer(D).

For the stability of the results, MR Egger Cochran's Q test showed no significant heterogeneity under the influence of SNPs for colon cancer, colitis, IBD, and CD (colon cancer: Q = 10.761, p = 0.869; colitis: Q = 11.482, p = 0.570; IBD: Q = 2.453, p = 0.117; and UC: Q = 0.623, P = 0.430) as illustrated in the funnel plot (Table 6, Figure 4A-D). The Funnel plots of ulcerative colitis, oesophageal cancer, gastric cancer, liver cell carcinoma, hepatic bile duct cancer, and pancreatic cancer were presented in Supplementary Figure 3. These results were also supported by the IVW method (Table 6). The MR-Egger method intercept p-values for colon cancer, colitis, IBD, and CD were 0.633, 0.952, 0.544, and 0.484, respectively (Table 6), all of which were greater than 0.05, indicating the absence of horizontal pleiotropy in the instrumental variables. This conclusion was further supported by the results of the MR-PRESSO global test method (Table 6). Additionally, leave-one-out sensitivity analyses were performed for IBD (Figure 5A), CD (Figure 5B), colitis (Figure 5C), and colon cancer (Figure 5D) to assess the effect of each SNP on the overall causal estimate. No significant change in the estimated causal effect was observed when individual SNPs were excluded (Figure 5). Leave-one-out sensitivity analyses of ulcerative colitis, oesophageal cancer, gastric cancer, liver cell carcinoma, hepatic bile duct cancer, and pancreatic cancer were presented in Supplementary Figure 4.

## Discussion

Glutamine, an abundant amino acid in the blood, plays diverse roles in the body, including gut protection and signaling in cancer cells 33-35. Previous observational studies have hinted at a link between glutamine and tumors, with reduced glutamine levels observed in colorectal cancer patients in clinical studies 5-8. Additionally, a study demonstrated the effectiveness of glutamine in controlling the progression of IBD and colitis 36-38. However, its role in gastrointestinal diseases and digestive tumors remains unclear. To address this gap, we conducted the first two-sample MR study to comprehensively assess the causal relationship between glutamine and the risk of developing digestive diseases in a European population. Through the selection of reliable SNPs as instrumental variables (IV), our findings suggest that genetically predicted glutamine levels are significantly associated with a reduced incidence of colon cancer, colitis, IBD, and its specific subtype Crohn's disease within a European population.

Initially, we identified instrumental variables representing exposure (circulating glutamine) from a large-scale UK Biobank cohort comprising 114,751 European individuals and analyzed them using the primary IVW methodology. This analysis revealed negative associations of circulating glutamine with colon cancer, colitis, and IBD. Subsequently, sensitivity analyses were conducted to examine heterogeneity, horizontal pleiotropy, and outliers when colon cancer, colitis, and IBD were utilized as outcomes.

Numerous previous studies have demonstrated that glutamine plays a role in promoting the development and progression of various cancers, including lung cancer 39, breast cancer 40 and colorectal cancer 41, owing to its involvement in cancer metabolism 35, 42-44. Nevertheless, conflicting findings exist, as some studies have reported adverse effects of glutamine supplementation 16, and elevated levels of glutamine were not found to enhance tumor growth in rat experiments 45. Recent investigations have revealed notable diversity in the glutamine requirements among different tumor types and even within different cell lines of the same tumor, such as luminal cells 46, 47. These variations arise due to the unique ways different cell types metabolize nutrients and generate energy, resulting in distinct nutritional demands. Such cell type-specific metabolic disparities are linked to numerous biological processes and facilitate symbiotic interactions between diverse cells and organisms. Furthermore, aside from its role in tumorigenesis, glutamine has been reported to have therapeutic effects on gastrointestinal disorders like colitis and IBD 37, 48, 49. However, the precise mechanism underlying this effect requires further elucidation.

In summary, there is evidence suggesting that circulating glutamine may reduce the risk of colon cancer, colitis, and Crohn's disease in IBD. While experimental studies have shown lower levels of circulating blood glutamine in colon cancer patients compared to normal tissues 5, 6, 10, and indicated a role for glutamine metabolism in promoting colon cancer progression 9, as well as its potential to alleviate colitis and IBD symptoms 36, 49, these findings do not conclusively establish glutamine as the direct cause of these conditions. For instance, inflammatory and cancerous cells both utilize glutamine for growth and energy, suggesting that glutamine may be a consequence rather than a cause in these contexts. Recent research has indicated that increasing glutamine levels can mitigate inflammation and enhance anti-tumor immune responses by alleviating endoplasmic reticulum stress and apoptosis in colitis 50, and serving as a crucial substrate for immune cell metabolism and inflammatory T-cell responses 11. Moreover, glutamine metabolism has been implicated in inhibiting cancer progression by promoting autophagy in tumors 51, 52. While these experimental mechanisms shed light on how glutamine might mitigate the incidence of colitis, IBD, and colon cancer, the current body of research on glutamine and intestinal diseases remains limited, necessitating further investigation to substantiate and expand upon these findings.

Our MR study boasts several advantages. Firstly, genetic testing for circulating glutamine helps eliminate potential confounders. Secondly, we derived correlations of genetic exposures from two independent GWAS datasets and employed various sensitivity analysis tests, all converging on the same conclusion. Thirdly, our study provides a theoretical basis for future prevention and treatment of colitis, IBD and colon cancer. However, this study still carries certain limitations. Firstly, all GWAS data used were sourced from individuals of European ancestry, with no available GWAS data from other ethnicities for validation. The theory of "population bottlenecks" suggests that different populations may harbor distinct genetic variations 53, thereby potentially limiting the generalizability of these findings to other racial populations globally. Secondly, due to dataset constraints, we couldn't explore whether the impact of circulating glutamine on intestinal disease varies by age or gender. Future studies should incorporate stratified MR analyses. Thirdly, we didn't validate across multiple datasets, including the utilization of GWAS data from various exposures corresponding to the same outcome or vice versa, which could enhance result reproducibility and confidence. Fourthly, all laboratory and clinical data analyzed in our present study were obtained from publicly available databases, and they have not undergone external experimental validation.

## Conclusions

In summary, this marks the inaugural MR investigation delving into the causal nexus between circulating glutamine levels and the vulnerability to bowel diseases and multiple digestive cancers, grounded in a European populace. Our MR analysis unveils a causal association between circulating glutamine and colon cancer, colitis, and IBD, indicating that elevated levels of circulating glutamine mitigate the risk of colitis, colon cancer, and IBD, while exhibiting no discernible impact on the risk of other digestive cancers. These revelations lay the groundwork for deeper dives into potential molecular mechanisms, epidemiological surveillance, and informed public health decision-making. Nonetheless, further large-scale studies are imperative to corroborate our findings and dissect the underlying mechanisms.

## Supplementary Material

Supplementary figures and tables.

## Figures and Tables

**Table 1:** Characteristic of the Glutamine-related genetic variants and their effects on IBD (3 SNPs)

SNP	Chr	Position	EA	SNPs-Glutamine			SNPs-IBD		
				β	SE	P value	β	SE	P value
rs3812316	7	73020337	G	0.0708251	0.006143	9.30E-31	-0.04927	0.015184	0.00117471
rs4237150	9	4290541	C	0.0330083	0.004206	4.20E-15	-0.02667	0.010485	0.0109602
rs838737	2	2.34E+08	A	-0.0234551	0.004162	1.70E-08	-0.00601	0.010467	0.565546

IBD inflammatory bowel disease; SNP single nucleotide polymorphism; Chr chromosome; EA effect allele; SE standard error

**Table 2:** Characteristic of the Glutamine-related genetic variants and their effects on CD (3 SNPs)

SNP	Chr	Position	EA	SNPs-Glutamine			SNPs-IBD		
				β	SE	P value	β	SE	P value
rs3812316	7	73020337	G	0.070825	0.006143	9.30E-31	-0.0778478	0.018572	2.77E-05
rs4237150	9	4290541	C	0.033008	0.004206	4.20E-15	-0.0348301	0.01268	0.006019
rs838737	2	234325052	A	-0.02346	0.004162	1.70E-08	0.00748466	0.012721	0.55629

CD Crohn's disease; SNP single nucleotide polymorphism; Chr chromosome; EA effect allele; SE standard error

**Table 3:** Characteristic of the Glutamine-related genetic variants and their effects on Colitis (15 SNPs)

SNP	Chr	Position	EA	SNPs-Glutamine			SNPs-Colitis		
				β	SE	P value	β	SE	P value
rs10849008	12	4302026	C	0.030089	0.004264	1.70E-12	-0.0000779584	0.000109	0.47
rs112081903	16	70014459	C	-0.02534	0.004514	2.00E-08	3.41E-05	0.000115	0.77
rs1260326	2	27730940	C	0.078942	0.004218	3.60E-78	-0.00015	0.000108	0.17
rs13094915	3	52507719	C	0.025648	0.004312	2.70E-09	-0.00015	0.00011	0.18
rs2039098	20	56112882	T	-0.02416	0.004195	8.40E-09	4.64E-05	0.000108	0.67
rs2168101	11	8255408	A	-0.06586	0.00461	2.70E-46	0.000141	0.000117	0.23
rs35007880	14	1.01E+08	T	0.033105	0.004167	2.00E-15	1.07E-05	0.000106	0.92
rs35261542	6	20683164	A	0.030213	0.004705	1.40E-10	0.000122	0.000114	0.28
rs4237150	9	4290085	C	0.033008	0.004206	4.20E-15	-0.000000911343	0.000107	0.99
rs4365129	12	47229840	T	-0.02657	0.004317	7.50E-10	0.000285	0.00011	0.0098
rs7147721	14	75232306	G	0.026091	0.004151	3.30E-10	7.95E-05	0.000106	0.450001
rs7925445	11	18398958	G	0.028546	0.004165	7.20E-12	-0.0000783641	0.000106	0.46
rs838737	2	2.34E+08	A	-0.02346	0.004162	1.70E-08	0.000129	0.000106	0.22
rs904538	17	25591429	A	0.027066	0.004139	6.20E-11	3.43E-05	0.000106	0.75
rs9482770	6	1.27E+08	C	-0.03234	0.004166	8.40E-15	7.47E-05	0.000106	0.48

SNP single nucleotide polymorphism; Chr chromosome; EA effect allele; SE standard error

**Table 4:** Characteristic of the Glutamine-related genetic variants and their effects on Colon cancer (19 SNPs)

SNP	Chr	Position	EA	SNPs-Glutamine			SNPs-Colon cancer		
				β	SE	P value	β	SE	P value
rs10849008	12	4302026	C	0.030089	0.004264	1.70E-12	-1.62E-05	0.000122	0.89
rs112081903	16	70014459	C	-0.02534	0.004514	2.00E-08	-7.06E-05	0.000129	0.58
rs11993225	8	1.34E+08	C	0.025967	0.004637	2.10E-08	5.85E-05	0.000133	0.66
rs1260326	2	27730940	C	0.078942	0.004218	3.60E-78	-0.000188606	0.00012	0.12
rs13094915	3	52507719	C	0.025648	0.004312	2.70E-09	7.37E-05	0.000123	0.55
rs1323320	6	56287985	A	-0.02588	0.004687	3.40E-08	-5.25E-07	0.000134	1
rs2039098	20	56112882	T	-0.02416	0.004195	8.40E-09	5.18E-05	0.00012	0.67
rs2168101	11	8255408	A	-0.06586	0.00461	2.70E-46	2.09E-05	0.000131	0.87
rs28362590	5	1.77E+08	T	-0.02621	0.004798	4.70E-08	1.98E-05	0.000137	0.88
rs35007880	14	1.01E+08	T	0.033105	0.004167	2.00E-15	-0.000263008	0.000119	0.027
rs35261542	6	20675792	A	0.030213	0.004705	1.40E-10	3.95E-05	0.000134	0.77
rs4237150	9	4290085	C	0.033008	0.004206	4.20E-15	-5.17E-05	0.00012	0.67
rs4365129	12	47229840	T	-0.02657	0.004317	7.50E-10	-0.00014963	0.000123	0.23
rs62182473	2	1.92E+08	T	-0.07072	0.00469	2.20E-51	0.000144747	0.000134	0.28
rs7147721	14	75232306	G	0.026091	0.004151	3.30E-10	-0.000111422	0.000118	0.35
rs7925445	11	18398958	G	0.028546	0.004165	7.20E-12	-4.52E-05	0.000119	0.7
rs838737	2	2.34E+08	A	-0.02346	0.004162	1.70E-08	0.000108859	0.000119	0.36
rs904538	17	25591429	A	0.027066	0.004139	6.20E-11	-8.32E-05	0.000118	0.48
rs9482770	6	1.27E+08	C	-0.03234	0.004166	8.40E-15	0.000175945	0.000119	0.14

SNP single nucleotide polymorphism; Chr chromosome; EA effect allele; SE standard error

**Table 5:** Effect estimates of the associations between Glutamine levels and colitis, IBD, and digestive system cancer in European populations.

Exposure GWAS ID	Outcome ID	Method	SNPs (N)	OR	95CI%	P value
Glutamine levels (ebi-a-GCST90092818)	Colitis					
	Colitis/not crohns or ulcerative colitis (ukb-b-3044)	MR Egger	15	0.998	0.995-1.002	0.383
		Weighted median	15	0.998	0.996-1.000	0.060
		IVW	15	0.998	0.997-1.000	**0.020**
		Simple mode	15	0.998	0.995-1.001	0.277
		Weighted mode	15	0.998	0.996-1.000	0.120
	IBD					
	Inflammatory bowel disease (ieu-a-294)	MR Egger	3	0.343	0.106-1.117	0.326
		Weighted median	3	0.491	0.341-0.706	1.26 E-04
		IVW	3	0.551	0.343-0.886	**0.014**
		Simple mode	3	0.471	0.300-0.739	0.082
		Weighted mode	3	0.484	0.332-0.706	0.064
	Ulcerative colitis (ieu-a-971)	MR Egger	5	0.619	0.042-9.184	0.751
		Weighted median	5	0.389	0.099-1.524	0.176
		IVW	5	0.508	0.163-1.586	0.244
		Simple mode	5	0.399	0.069-2.303	0.362
		Weighted mode	5	0.365	0.076-1.754	0.277
	Crohn's disease (ieu-a-12)	MR Egger	3	0.240	0.096-0.603	2.02E-01
		Weighted median	3	0.341	0.220-0.526	1.24E-06
		IVW	3	0.375	0.253-0.557	**1.11E-06**
		Simple mode	3	0.341	0.197-0.589	6.11E-02
		Weighted mode	3	0.337	0.207-0.550	4.90E-02
	Digestive system cancer					
	Oesophageal cancer (ieu-b-4960)	MR Egger	37	0.999	0.998-1.001	0.327
		Weighted median	37	0.999	0.998-1.000	0.269
		IVW	37	1.000	0.999-1.001	0.566
		Simple mode	37	0.999	0.997-1.001	0.252
		Weighted mode	37	1.000	0.999-1.000	0.356
	Gastric cancer (bbj-a-119)	MR Egger	30	1.078	0.864-1.345	0.509
		Weighted median	30	0.993	0.833-1.184	0.940
		IVW	30	0.966	0.832-1.121	0.648
		Simple mode	30	1.007	0.756-1.340	0.963
		Weighted mode	30	1.007	0.851-1.192	0.937
	Liver cell carcinoma (ieu-b-4953)	MR Egger	28	1.000	0.999-1.000	0.098
		Weighted median	28	1.000	0.999-1.000	0.297
		IVW	28	1.000	0.999-1.000	0.397
		Simple mode	28	1.000	0.999-1.002	0.539
		Weighted mode	28	1.000	0.999-1.000	0.355
	Hepatic bile duct cancer (ebi-a-GCST90018583)	MR Egger	32	0.569	0.270-1.199	0.149
		Weighted median	32	0.769	0.382-1.548	0.461
		IVW	32	0.819	0.499-1.344	0.430
		Simple mode	32	0.952	0.340-2.662	0.926
		Weighted mode	32	0.707	0.394-1.270	0.255
	Pancreatic cancer (ebi-a-GCST90018893)	MR Egger	41	1.175	0.832-1.659	0.366
		Weighted median	41	0.863	0.632-1.180	0.357
		IVW	41	1.130	0.897-1.423	0.301
		Simple mode	41	1.892	0.986-3.629	0.062
		Weighted mode	41	0.972	0.708-1.336	0.863
	Colon cancer/sigmoid cancer (ukb-b-20145)	MR Egger	19	0.998	0.994-1.001	0.184
		Weighted median	19	0.998	0.996-1.000	0.053
		IVW	19	0.998	0.997-1.000	**0.027**
		Simple mode	19	0.999	0.995-1.002	0.410
		Weighted mode	19	0.998	0.996-1.001	0.142

**Table 6:** Sensitivity analyses between Glutamine levels and colitis, IBD, and digestive system cancers in European populations.

Exposure GWAS ID	Outcome GWAS ID	Method	Heterogeneity Q/P value	Pleiotropy/P value
Glutamine levels (ebi-a-GCST90092818)	Colitis			
	Colitis/not crohns or ulcerative colitis (ukb-b-3044)	MR Egger	11.482/0.570	
		Inverse variance weighted	11.486/0.647	
		MR-PRESSO global test		0.758
		Intercept from MR Egger regression analysis		0.952
	IBD			
	Inflammatory bowel disease (ieu-a-294)	MR Egger	2.453/0.117	
		Inverse variance weighted	4.310/0.116	
		MR-PRESSO global test		
		Intercept from MR Egger regression analysis		0.544
	Ulcerative colitis (ieu-a-971)	MR Egger	0.774/0.856	
		Inverse variance weighted	0.799/0.938	
		MR-PRESSO global test		
		Intercept from MR Egger regression analysis		0.884
	Crohn's disease (ieu-a-12)	MR Egger	0.623/0.430	
		Inverse variance weighted	1.730/0.421	
		MR-PRESSO global test		
		Intercept from MR Egger regression analysis		0.484
	Digestive system cancer			
	Oesophageal cancer (ieu-b-4960)	MR Egger	38.876/0.299	
		Inverse variance weighted	39.626/0.311	
		MR-PRESSO global test		0.379
		Intercept from MR Egger regression analysis		0.417
	Gastric cancer (bbj-a-119)	MR Egger	34.685/0.179	
		Inverse variance weighted	36.794/0.152	
		MR-PRESSO global test		0.203
		Intercept from MR Egger regression analysis		0.179
	Liver cell carcinoma (ieu-b-4953)	MR Egger	20.742/0.755	
		Inverse variance weighted	23.147/0.677	
		MR-PRESSO global test		0.721
		Intercept from MR Egger regression analysis		0.133
	Hepatic bile duct cancer (ebi-a-GCST90018583)	MR Egger	16.583/0.977	
		Inverse variance weighted	18.228/0.967	
		MR-PRESSO global test		0.971
		Intercept from MR Egger regression analysis		0.210
	Pancreatic cancer (ebi-a-GCST90018893)	MR Egger	36.039/0.606	
		Inverse variance weighted	36.128/0.645	
		MR-PRESSO global test		0.497
		Intercept from MR Egger regression analysis		0.766
	colon cancer/sigmoid cancer (ukb-b-20145)	MR Egger	10.761/0.869	
		Inverse variance weighted	10.998/0.894	
		MR-PRESSO global test		0.880
		Intercept from MR Egger regression analysis		0.633

## References

[1] Darmaun D, Matthews DE, Bier DM (1986). Glutamine and glutamate kinetics in humans. Am J Physiol.

[2] Curi R, Lagranha CJ, Doi SQ, Sellitti DF, Procopio J, Pithon-Curi TC (2005). Molecular mechanisms of glutamine action. J Cell Physiol.

[3] Cruzat V, Macedo Rogero M, Noel Keane K, Curi R, Newsholme P (2018). Glutamine: Metabolism and Immune Function, Supplementation and Clinical Translation. Nutrients.

[4] Reeds PJ, Burrin DG (2001). Glutamine and the bowel. The Journal of nutrition.

[5] Tan B, Qiu Y, Zou X, Chen T, Xie G, Cheng Y (2013). Metabonomics identifies serum metabolite markers of colorectal cancer. Journal of proteome research.

[6] Zhu J, Djukovic D, Deng L, Gu H, Himmati F, Chiorean EG (2014). Colorectal cancer detection using targeted serum metabolic profiling. J Proteome Res.

[7] Bertini I, Cacciatore S, Jensen BV, Schou JV, Johansen JS, Kruhøffer M (2012). Metabolomic NMR fingerprinting to identify and predict survival of patients with metastatic colorectal cancer. Cancer Res.

[8] Kim H (2011). Glutamine as an immunonutrient. Yonsei Med J.

[9] Sun H, Zhang C, Zheng Y, Liu C, Wang X, Cong X (2022). Glutamine deficiency promotes recurrence and metastasis in colorectal cancer through enhancing epithelial-mesenchymal transition. Journal of translational medicine.

[10] Ling HH, Pan YP, Fan CW, Tseng WK, Huang JS, Wu TH (2019). Clinical Significance of Serum Glutamine Level in Patients with Colorectal Cancer. Nutrients.

[11] Yang T, Yan X, Cao Y, Bao T, Li G, Gu S (2021). Meta-analysis of Glutamine on Immune Function and Post-Operative Complications of Patients With Colorectal Cancer. Frontiers in nutrition.

[12] Xiong K, Li G, Zhang Y, Bao T, Li P, Yang X (2023). Effects of glutamine on plasma protein and inflammation in postoperative patients with colorectal cancer: a meta-analysis of randomized controlled trials. International journal of colorectal disease.

[13] Narula N, Dhillon A, Zhang D, Sherlock ME, Tondeur M, Zachos M (2018). Enteral nutritional therapy for induction of remission in Crohn's disease. The Cochrane database of systematic reviews.

[14] Yan S, Hui Y, Li J, Xu X, Li Q, Wei H (2020). Glutamine relieves oxidative stress through PI3K/Akt signaling pathway in DSS-induced ulcerative colitis mice. Iranian journal of basic medical sciences.

[15] Kalaany NY (2024). Glutamine analogs for pancreatic cancer therapy. Nature cancer.

[16] Holecek M (2013). Side effects of long-term glutamine supplementation. JPEN Journal of parenteral and enteral nutrition.

[17] Davey Smith G, Hemani G (2014). Mendelian randomization: genetic anchors for causal inference in epidemiological studies. Human molecular genetics.

[18] Lee K, Lim CY (2019). Mendelian Randomization Analysis in Observational Epidemiology. Journal of lipid and atherosclerosis.

[19] Zhang K, Liang H (2023). Genetic estimation of correlations between circulating glutamine and cancer. American journal of cancer research.

[20] Richardson TG, Leyden GM, Wang Q, Bell JA, Elsworth B, Davey Smith G (2022). Characterising metabolomic signatures of lipid-modifying therapies through drug target mendelian randomisation. PLoS biology.

[21] Auton A, Brooks LD, Durbin RM, Garrison EP, Kang HM, Korbel JO (2015). A global reference for human genetic variation. Nature.

[22] Sudlow C, Gallacher J, Allen N, Beral V, Burton P, Danesh J (2015). UK biobank: an open access resource for identifying the causes of a wide range of complex diseases of middle and old age. PLoS medicine.

[23] Liu JZ, van Sommeren S, Huang H, Ng SC, Alberts R, Takahashi A (2015). Association analyses identify 38 susceptibility loci for inflammatory bowel disease and highlight shared genetic risk across populations. Nat Genet.

[24] Sakaue S, Kanai M, Tanigawa Y, Karjalainen J, Kurki M, Koshiba S (2021). A cross-population atlas of genetic associations for 220 human phenotypes. Nat Genet.

[25] Zhou W, Zhao Z, Nielsen JB, Fritsche LG, LeFaive J, Gagliano Taliun SA (2020). Scalable generalized linear mixed model for region-based association tests in large biobanks and cohorts. Nat Genet.

[26] Yavorska OO, Burgess S (2017). MendelianRandomization: an R package for performing Mendelian randomization analyses using summarized data. International journal of epidemiology.

[27] Larsson SC, Traylor M, Malik R, Dichgans M, Burgess S, Markus HS (2017). Modifiable pathways in Alzheimer's disease: Mendelian randomisation analysis. BMJ (Clinical research ed).

[28] Larsson SC, Burgess S (2022). Appraising the causal role of smoking in multiple diseases: A systematic review and meta-analysis of Mendelian randomization studies. EBioMedicine.

[29] Burgess S, Butterworth A, Thompson SG (2013). Mendelian randomization analysis with multiple genetic variants using summarized data. Genetic epidemiology.

[30] Greco MF, Minelli C, Sheehan NA, Thompson JR (2015). Detecting pleiotropy in Mendelian randomisation studies with summary data and a continuous outcome. Statistics in medicine.

[31] Bowden J, Davey Smith G, Burgess S (2015). Mendelian randomization with invalid instruments: effect estimation and bias detection through Egger regression. International journal of epidemiology.

[32] Verbanck M, Chen CY, Neale B, Do R (2018). Detection of widespread horizontal pleiotropy in causal relationships inferred from Mendelian randomization between complex traits and diseases. Nat Genet.

[33] Hensley CT, Wasti AT, DeBerardinis RJ (2013). Glutamine and cancer: cell biology, physiology, and clinical opportunities. J Clin Invest.

[34] Altman BJ, Stine ZE, Dang CV (2016). From Krebs to clinic: glutamine metabolism to cancer therapy. Nature reviews Cancer.

[35] Leone RD, Zhao L, Englert JM, Sun IM, Oh MH, Sun IH (2019). Glutamine blockade induces divergent metabolic programs to overcome tumor immune evasion. Science.

[36] Akobeng AK, Elawad M, Gordon M (2016). Glutamine for induction of remission in Crohn's disease. The Cochrane database of systematic reviews.

[37] Jeong SY, Im YN, Youm JY, Lee HK, Im SY (2018). l-Glutamine Attenuates DSS-Induced Colitis via Induction of MAPK Phosphatase-1. Nutrients.

[38] Severo JS, da Silva Barros VJ, Alves da Silva AC, Luz Parente JM, Lima MM, Moreira Lima A (2021). Effects of glutamine supplementation on inflammatory bowel disease: A systematic review of clinical trials. Clinical nutrition ESPEN.

[39] Best SA, Gubser PM, Sethumadhavan S, Kersbergen A, Negrón Abril YL, Goldford J (2022). Glutaminase inhibition impairs CD8 T cell activation in STK11-/Lkb1-deficient lung cancer. Cell Metab.

[40] Edwards DN, Ngwa VM, Raybuck AL, Wang S, Hwang Y, Kim LC (2021). Selective glutamine metabolism inhibition in tumor cells improves antitumor T lymphocyte activity in triple-negative breast cancer. J Clin Invest.

[41] Hao Y, Samuels Y, Li Q, Krokowski D, Guan BJ, Wang C (2016). Oncogenic PIK3CA mutations reprogram glutamine metabolism in colorectal cancer. Nature communications.

[42] Cluntun AA, Lukey MJ, Cerione RA, Locasale JW (2017). Glutamine Metabolism in Cancer: Understanding the Heterogeneity. Trends in cancer.

[43] Kodama M, Oshikawa K, Shimizu H, Yoshioka S, Takahashi M, Izumi Y (2020). A shift in glutamine nitrogen metabolism contributes to the malignant progression of cancer. Nature communications.

[44] Yang WH, Qiu Y, Stamatatos O, Janowitz T, Lukey MJ (2021). Enhancing the Efficacy of Glutamine Metabolism Inhibitors in Cancer Therapy. Trends in cancer.

[45] Davidson SM, Papagiannakopoulos T, Olenchock BA, Heyman JE, Keibler MA, Luengo A (2016). Environment Impacts the Metabolic Dependencies of Ras-Driven Non-Small Cell Lung Cancer. Cell Metab.

[46] Kung HN, Marks JR, Chi JT (2011). Glutamine synthetase is a genetic determinant of cell type-specific glutamine independence in breast epithelia. PLoS genetics.

[47] Timmerman LA, Holton T, Yuneva M, Louie RJ, Padró M, Daemen A (2013). Glutamine sensitivity analysis identifies the xCT antiporter as a common triple-negative breast tumor therapeutic target. Cancer Cell.

[48] Elia M, Lunn PG (1997). The use of glutamine in the treatment of gastrointestinal disorders in man. Nutrition (Burbank, Los Angeles County, Calif).

[49] Kaya E, Gür ES, Ozgüç H, Bayer A, Tokyay R (1999). L-glutamine enemas attenuate mucosal injury in experimental colitis. Diseases of the colon and rectum.

[50] Crespo I, San-Miguel B, Prause C, Marroni N, Cuevas MJ, González-Gallego J (2012). Glutamine treatment attenuates endoplasmic reticulum stress and apoptosis in TNBS-induced colitis. PLoS One.

[51] Bodineau C, Tomé M, Murdoch PDS, Durán RV (2022). Glutamine, MTOR and autophagy: a multiconnection relationship. Autophagy.

[52] Mukha A, Kahya U, Dubrovska A (2021). Targeting glutamine metabolism and autophagy: the combination for prostate cancer radiosensitization. Autophagy.

[53] Kim MS, Patel KP, Teng AK, Berens AJ, Lachance J (2018). Genetic disease risks can be misestimated across global populations. Genome Biol.

