# Supplementary material for: Causal effects of circulating glutamine on colitis, IBD, and digestive system cancers: a Mendelian randomisation study
Source: J Cancer. 2024 May 20;15(12):3738–49. doi: 10.7150/jca.96085 (PMC11190753; doi:10.7150/jca.96085)
Supplement: Supplementary file 1 — Supplementary figures and tables. [file jcav15p3738s1.zip › Supplementary Table 1.docx]

Supplementary Table1. Specific information on instrumental variables.

| chr.  exposure | pos.exposure | beta.  exposure | se.  exposure | pval.  exposure | id.  exposure | SNP | effect_  allele.  exposure | other_  allele.  exposure | eaf.  exposure | F statistic |
| --- | --- | --- | --- | --- | --- | --- | --- | --- | --- | --- |
| 1 | 214150821 | 0.156 | 0.011 | 4.300E-43 | ebi-a-GCST90092818 | rs79687284 | C | G | 0.035 | 189.402 |
| 2 | 27730940 | 0.079 | 0.004 | 3.600E-78 | ebi-a-GCST90092818 | rs1260326 | C | T | 0.604 | 350.337 |
| 2 | 211418890 | 0.024 | 0.004 | 2.400E-08 | ebi-a-GCST90092818 | rs4673538 | T | C | 0.370 | 31.145 |
| 2 | 234325052 | (0.023) | 0.004 | 1.700E-08 | ebi-a-GCST90092818 | rs838737 | A | G | 0.565 | 31.757 |
| 2 | 191740996 | (0.071) | 0.005 | 2.200E-51 | ebi-a-GCST90092818 | rs62182473 | T | C | 0.265 | 227.394 |
| 3 | 39191335 | (0.030) | 0.005 | 3.000E-09 | ebi-a-GCST90092818 | rs1274961 | C | T | 0.783 | 35.199 |
| 3 | 52532291 | 0.024 | 0.004 | 1.100E-08 | ebi-a-GCST90092818 | rs34736619 | C | G | 0.379 | 32.627 |
| 3 | 160258869 | (0.042) | 0.004 | 4.000E-24 | ebi-a-GCST90092818 | rs56156703 | T | A | 0.498 | 102.645 |
| 5 | 176731452 | (0.026) | 0.005 | 4.700E-08 | ebi-a-GCST90092818 | rs28362590 | T | G | 0.754 | 29.830 |
| 6 | 127443092 | (0.032) | 0.004 | 8.400E-15 | ebi-a-GCST90092818 | rs9482770 | C | T | 0.449 | 60.240 |
| 6 | 131877661 | (0.036) | 0.005 | 5.901E-13 | ebi-a-GCST90092818 | rs2608977 | C | T | 0.223 | 51.875 |
| 6 | 56287985 | (0.026) | 0.005 | 3.400E-08 | ebi-a-GCST90092818 | rs1323320 | A | G | 0.263 | 30.483 |
| 6 | 20675792 | 0.030 | 0.005 | 1.400E-10 | ebi-a-GCST90092818 | rs35261542 | A | C | 0.261 | 41.233 |
| 7 | 73020337 | 0.071 | 0.006 | 9.300E-31 | ebi-a-GCST90092818 | rs3812316 | G | C | 0.129 | 132.946 |
| 7 | 1885600 | 0.030 | 0.005 | 1.100E-09 | ebi-a-GCST90092818 | rs58673065 | G | A | 0.229 | 37.159 |
| 8 | 17419461 | 0.117 | 0.013 | 1.400E-20 | ebi-a-GCST90092818 | rs56335308 | A | G | 0.027 | 86.480 |
| 8 | 134335352 | 0.026 | 0.005 | 2.100E-08 | ebi-a-GCST90092818 | rs11993225 | C | A | 0.270 | 31.361 |
| 8 | 17374777 | 0.041 | 0.005 | 3.700E-17 | ebi-a-GCST90092818 | rs2720586 | T | C | 0.767 | 70.908 |
| 8 | 126500031 | 0.032 | 0.004 | 7.300E-14 | ebi-a-GCST90092818 | rs28601761 | G | C | 0.420 | 55.996 |
| 9 | 22139220 | (0.048) | 0.008 | 2.200E-09 | ebi-a-GCST90092818 | rs10811663 | A | G | 0.086 | 35.807 |
| 9 | 4290085 | 0.033 | 0.004 | 4.200E-15 | ebi-a-GCST90092818 | rs4237150 | C | G | 0.403 | 61.592 |
| 10 | 114783586 | (0.024) | 0.004 | 4.900E-09 | ebi-a-GCST90092818 | rs6585204 | G | C | 0.466 | 34.223 |
| 10 | 88820592 | (0.051) | 0.009 | 1.500E-08 | ebi-a-GCST90092818 | rs17096421 | T | A | 0.056 | 32.084 |
| 10 | 22926227 | 0.057 | 0.010 | 4.700E-08 | ebi-a-GCST90092818 | rs1750768 | T | C | 0.042 | 29.852 |
| 10 | 99385055 | 0.027 | 0.004 | 1.300E-10 | ebi-a-GCST90092818 | rs6584138 | C | A | 0.556 | 41.324 |
| 10 | 99359412 | 0.104 | 0.005 | 8.800E-83 | ebi-a-GCST90092818 | rs7078003 | T | C | 0.178 | 371.510 |
| 11 | 8255408 | (0.066) | 0.005 | 2.700E-46 | ebi-a-GCST90092818 | rs2168101 | A | C | 0.308 | 204.077 |
| 11 | 18398958 | 0.029 | 0.004 | 7.199E-12 | ebi-a-GCST90092818 | rs7925445 | G | A | 0.556 | 46.977 |
| 11 | 74109553 | 0.079 | 0.010 | 2.600E-14 | ebi-a-GCST90092818 | rs78431863 | T | C | 0.041 | 58.022 |
| 12 | 4301876 | 0.030 | 0.004 | 1.700E-12 | ebi-a-GCST90092818 | rs10849008 | C | T | 0.381 | 49.795 |
| 12 | 121401846 | (0.024) | 0.004 | 4.000E-08 | ebi-a-GCST90092818 | rs7966322 | T | C | 0.540 | 30.131 |
| 12 | 57069929 | 0.037 | 0.004 | 1.100E-16 | ebi-a-GCST90092818 | rs56183434 | T | G | 0.667 | 68.798 |
| 12 | 56888701 | 0.086 | 0.008 | 5.400E-28 | ebi-a-GCST90092818 | rs141768399 | T | C | 0.085 | 120.320 |
| 12 | 57304853 | (0.120) | 0.022 | 2.300E-08 | ebi-a-GCST90092818 | rs117001881 | A | G | 0.010 | 31.225 |
| 12 | 47160241 | (0.079) | 0.012 | 2.800E-11 | ebi-a-GCST90092818 | rs117433039 | C | T | 0.032 | 44.299 |
| 12 | 56848079 | 0.085 | 0.013 | 1.400E-11 | ebi-a-GCST90092818 | rs74641138 | T | C | 0.028 | 45.635 |
| 12 | 59647771 | (0.036) | 0.006 | 2.800E-08 | ebi-a-GCST90092818 | rs112148652 | A | G | 0.118 | 30.847 |
| 12 | 56936263 | 0.098 | 0.017 | 3.900E-09 | ebi-a-GCST90092818 | rs11835668 | A | T | 0.025 | 34.654 |
| 12 | 59990767 | 0.080 | 0.007 | 2.300E-32 | ebi-a-GCST90092818 | rs17122673 | C | T | 0.107 | 140.255 |
| 12 | 56860020 | (0.250) | 0.005 | 1.00E-200 | ebi-a-GCST90092818 | rs2939302 | A | G | 0.183 | 2201.751 |
| 12 | 47349020 | (0.029) | 0.004 | 7.000E-12 | ebi-a-GCST90092818 | rs11183671 | C | T | 0.561 | 47.033 |
| 12 | 47198856 | 0.134 | 0.008 | 3.100E-60 | ebi-a-GCST90092818 | rs113674212 | A | C | 0.068 | 267.996 |
| 12 | 57426973 | 0.084 | 0.010 | 3.100E-18 | ebi-a-GCST90092818 | rs697223 | C | A | 0.952 | 75.805 |
| 14 | 100796401 | 0.033 | 0.004 | 2.400E-15 | ebi-a-GCST90092818 | rs3736951 | T | C | 0.513 | 62.698 |
| 14 | 21532312 | 0.039 | 0.006 | 8.900E-12 | ebi-a-GCST90092818 | rs12881221 | T | C | 0.156 | 46.549 |
| 14 | 75186010 | 0.026 | 0.004 | 3.300E-10 | ebi-a-GCST90092818 | rs7147721 | G | A | 0.462 | 39.514 |
| 14 | 94844947 | 0.091 | 0.015 | 8.900E-10 | ebi-a-GCST90092818 | rs28929474 | T | C | 0.020 | 37.559 |
| 14 | 21492229 | 0.167 | 0.021 | 1.500E-15 | ebi-a-GCST90092818 | rs1998848 | A | G | 0.011 | 63.641 |
| 16 | 70014459 | (0.025) | 0.005 | 2.000E-08 | ebi-a-GCST90092818 | rs112081903 | C | T | 0.299 | 31.517 |
| 17 | 25591429 | 0.027 | 0.004 | 6.200E-11 | ebi-a-GCST90092818 | rs904538 | A | C | 0.463 | 42.770 |
| 20 | 56112882 | (0.024) | 0.004 | 8.400E-09 | ebi-a-GCST90092818 | rs2039098 | T | C | 0.597 | 33.177 |
| 22 | 44324730 | 0.032 | 0.005 | 1.000E-10 | ebi-a-GCST90092818 | rs738408 | T | C | 0.217 | 41.773 |
