# Supplementary material for: Causal effects of circulating glutamine on colitis, IBD, and digestive system cancers: a Mendelian randomisation study
Source: J Cancer. 2024 May 20;15(12):3738–49. doi: 10.7150/jca.96085 (PMC11190753; doi:10.7150/jca.96085)
Supplement: Supplementary file 1 — Supplementary figures and tables. [file jcav15p3738s1.zip › Supplementary Table 2-7.docx]

Table S2. Characteristic of the Glutamine-related genetic variants and their effects on UC (5 SNPs)

| SNP | Chr | Position | EA | SNPs-Glutamine | | | SNPs-UC | | |
| --- | --- | --- | --- | --- | --- | --- | --- | --- | --- |
|  |  |  |  | β | SE | P value | β | SE | P value |
| rs1260326 | 2 | 27730940 | C | 0.078942 | 0.004218 | 3.60E-78 | -0.08217 | 0.069587 | 0.237751 |
| rs35261542 | 6 | 20675792 | A | 0.030213 | 0.004705 | 1.40E-10 | -0.02421 | 0.068535 | 0.723872 |
| rs3812316 | 7 | 73020337 | G | 0.070825 | 0.006143 | 9.30E-31 | -0.06519 | 0.101507 | 0.520735 |
| rs62182473 | 2 | 1.92E+08 | T | -0.07072 | 0.00469 | 2.20E-51 | -0.00802 | 0.075644 | 0.915607 |
| rs838737 | 2 | 2.34E+08 | A | -0.02346 | 0.004162 | 1.70E-08 | 0.030997 | 0.066948 | 0.64337 |

UC ulcerative colitis; SNP single nucleotide polymorphism; Chr chromosome; EA effect allele; SE standard error

Table S3. Characteristic of the Glutamine-related genetic variants and their effects on Oesophageal cancer (37 SNPs)

| SNP | Chr | Position | EA | SNPs-Glutamine | | | SNPs- Oesophageal cancer | | |
| --- | --- | --- | --- | --- | --- | --- | --- | --- | --- |
|  |  |  |  | β | SE | P value | β | SE | P value |
| rs10811663 | 9 | 22139220 | A | -0.04815 | 0.008046 | 2.20E-09 | 0.000110415 | 0.000201 | 0.58 |
| rs10849008 | 12 | 4301876 | C | 0.030089 | 0.004264 | 1.70E-12 | 0.000166294 | 0.000106 | 0.12 |
| rs112081903 | 16 | 70014459 | C | -0.02534 | 0.004514 | 2.00E-08 | -0.00013928 | 0.000113 | 0.22 |
| rs113674212 | 12 | 47198856 | A | 0.134105 | 0.008192 | 3.10E-60 | -6.36E-05 | 0.000205 | 0.760001 |
| rs11386117 | 10 | 1.15E+08 | CA | -0.02421 | 0.004133 | 4.70E-09 | -0.00012571 | 0.000103 | 0.22 |
| rs11422720 | 6 | 1.32E+08 | GC | -0.03616 | 0.00498 | 3.80E-13 | 0.000226493 | 0.000124 | 0.068 |
| rs11993225 | 8 | 1.34E+08 | C | 0.025967 | 0.004637 | 2.10E-08 | 0.000343104 | 0.000116 | 0.0031 |
| rs1260326 | 2 | 27730940 | C | 0.078942 | 0.004218 | 3.60E-78 | -4.90E-06 | 0.000105 | 0.96 |
| rs1274961 | 3 | 39191335 | C | -0.02967 | 0.005001 | 3.00E-09 | 9.06E-05 | 0.000125 | 0.47 |
| rs13094915 | 3 | 52507719 | C | 0.025648 | 0.004312 | 2.70E-09 | 0.00012254 | 0.000107 | 0.25 |
| rs1323320 | 6 | 56287985 | A | -0.02588 | 0.004687 | 3.40E-08 | 6.86E-05 | 0.000117 | 0.56 |
| rs142525555 | 12 | 57232374 | AT | -0.06146 | 0.009776 | 3.20E-10 | 0.000125557 | 0.000245 | 0.61 |
| rs17096421 | 10 | 88820592 | T | -0.05138 | 0.00907 | 1.50E-08 | -5.11E-06 | 0.000226 | 0.98 |
| rs1750768 | 10 | 22926227 | T | 0.056523 | 0.010345 | 4.70E-08 | 0.000216842 | 0.000257 | 0.4 |
| rs2039098 | 20 | 56115153 | T | -0.02416 | 0.004195 | 8.40E-09 | 7.59E-05 | 0.000104 | 0.46 |
| rs2168101 | 11 | 8255408 | A | -0.06586 | 0.00461 | 2.70E-46 | 8.84E-05 | 0.000115 | 0.44 |
| rs2657879 | 12 | 56865338 | G | -0.25113 | 0.005326 | 1.00E-200 | 0.000146182 | 0.000133 | 0.27 |
| rs28362590 | 5 | 1.77E+08 | T | -0.02621 | 0.004798 | 4.70E-08 | -9.14E-06 | 0.00012 | 0.94 |
| rs28929474 | 14 | 94844947 | T | 0.090546 | 0.014775 | 8.90E-10 | -0.00063712 | 0.000371 | 0.086 |
| rs35007880 | 14 | 1.01E+08 | T | 0.033105 | 0.004167 | 2.00E-15 | -3.49E-05 | 0.000104 | 0.74 |
| rs35213094 | 12 | 59945417 | A | 0.065283 | 0.005919 | 2.80E-28 | -0.00020914 | 0.000148 | 0.16 |
| rs35261542 | 6 | 20675792 | A | 0.030213 | 0.004705 | 1.40E-10 | -9.94E-05 | 0.000117 | 0.4 |
| rs3812316 | 7 | 73020337 | G | 0.070825 | 0.006143 | 9.30E-31 | 9.42E-05 | 0.000154 | 0.54 |
| rs4237150 | 9 | 4290541 | C | 0.033008 | 0.004206 | 4.20E-15 | -1.73E-05 | 0.000105 | 0.87 |
| rs4365129 | 12 | 47237515 | T | -0.02657 | 0.004317 | 7.50E-10 | 4.89E-06 | 0.000108 | 0.96 |
| rs56335308 | 8 | 17419461 | A | 0.117482 | 0.012633 | 1.40E-20 | 0.000275209 | 0.000317 | 0.39 |
| rs58673065 | 7 | 1885600 | G | 0.029884 | 0.004902 | 1.10E-09 | -7.41E-05 | 0.000123 | 0.55 |
| rs62182473 | 2 | 1.92E+08 | T | -0.07072 | 0.00469 | 2.20E-51 | -7.92E-05 | 0.000117 | 0.5 |
| rs7078003 | 10 | 99359412 | T | 0.104082 | 0.0054 | 8.80E-83 | 0.000168135 | 0.000135 | 0.21 |
| rs7147721 | 14 | 75232306 | G | 0.026091 | 0.004151 | 3.30E-10 | 8.98E-05 | 0.000104 | 0.39 |
| rs738408 | 22 | 44324730 | T | 0.03237 | 0.005008 | 1.00E-10 | -5.47E-05 | 0.000125 | 0.66 |
| rs78431863 | 11 | 74109553 | T | 0.079422 | 0.010427 | 2.60E-14 | -0.0001257 | 0.000261 | 0.630001 |
| rs7925445 | 11 | 18398958 | G | 0.028546 | 0.004165 | 7.20E-12 | -3.90E-05 | 0.000104 | 0.709999 |
| rs79687284 | 1 | 2.14E+08 | C | 0.155571 | 0.011304 | 4.30E-43 | 2.14E-05 | 0.000282 | 0.94 |
| rs838737 | 2 | 2.34E+08 | A | -0.02346 | 0.004162 | 1.70E-08 | -8.02E-05 | 0.000104 | 0.44 |
| rs904538 | 17 | 25595818 | A | 0.027066 | 0.004139 | 6.20E-11 | -0.0002282 | 0.000104 | 0.028 |
| rs9482770 | 6 | 1.27E+08 | C | -0.03234 | 0.004166 | 8.40E-15 | -7.37E-05 | 0.000104 | 0.48 |

SNP single nucleotide polymorphism; Chr chromosome; EA effect allele; SE standard error

Table S4. Characteristic of the Glutamine-related genetic variants and their effects on Gastric cancer (30 SNPs)

| SNP | Chr | Position | EA | SNPs-Glutamine | | | SNPs- Gastric cancer | | |
| --- | --- | --- | --- | --- | --- | --- | --- | --- | --- |
|  |  |  |  | β | SE | P value | β | SE | P value |
| rs10811663 | 9 | 22139220 | A | -0.04815 | 0.008046 | 2.20E-09 | 0.021285 | 0.026066 | 0.41418 |
| rs10849008 | 12 | 4301876 | C | 0.030089 | 0.004264 | 1.70E-12 | -0.00823 | 0.018633 | 0.658792 |
| rs113674212 | 12 | 47198856 | A | 0.134105 | 0.008192 | 3.10E-60 | 0.009005 | 0.039942 | 0.821634 |
| rs11386117 | 10 | 1.15E+08 | CA | -0.02421 | 0.004133 | 4.70E-09 | 0.004008 | 0.036743 | 0.913144 |
| rs11422720 | 6 | 1.32E+08 | GC | -0.03616 | 0.00498 | 3.80E-13 | 0.002063 | 0.019101 | 0.913971 |
| rs1260326 | 2 | 27730940 | C | 0.078942 | 0.004218 | 3.60E-78 | 0.001146 | 0.018158 | 0.949675 |
| rs1274961 | 3 | 39191335 | C | -0.02967 | 0.005001 | 3.00E-09 | 0.050294 | 0.209757 | 0.810505 |
| rs1323320 | 6 | 56287985 | A | -0.02588 | 0.004687 | 3.40E-08 | 0.027776 | 0.018299 | 0.12904 |
| rs13396296 | 2 | 2.11E+08 | C | -0.02419 | 0.004342 | 2.50E-08 | -0.01553 | 0.020534 | 0.449529 |
| rs1750768 | 10 | 22965448 | T | 0.056523 | 0.010345 | 4.70E-08 | 1.4413 | 1.57935 | 0.361458 |
| rs1998848 | 14 | 21492229 | A | 0.166954 | 0.020928 | 1.50E-15 | 0.020966 | 0.028955 | 0.469019 |
| rs2039098 | 20 | 56112882 | T | -0.02416 | 0.004195 | 8.40E-09 | -0.00587 | 0.018731 | 0.753966 |
| rs2168101 | 11 | 8255408 | A | -0.06586 | 0.00461 | 2.70E-46 | -0.01299 | 0.023267 | 0.576765 |
| rs2657879 | 12 | 56865338 | G | -0.25113 | 0.005326 | 1.00E-200 | 0.012725 | 0.02939 | 0.665048 |
| rs28362590 | 5 | 1.77E+08 | T | -0.02621 | 0.004798 | 4.70E-08 | -0.00215 | 0.017998 | 0.904797 |
| rs35007880 | 14 | 1.01E+08 | T | 0.033105 | 0.004167 | 2.00E-15 | -0.02862 | 0.026101 | 0.272913 |
| rs35213094 | 12 | 59948824 | A | 0.065283 | 0.005919 | 2.80E-28 | -0.04329 | 0.310213 | 0.889013 |
| rs35261542 | 6 | 20675792 | A | 0.030213 | 0.004705 | 1.40E-10 | -0.0813 | 0.01837 | 9.62E-06 |
| rs3812316 | 7 | 73020337 | G | 0.070825 | 0.006143 | 9.30E-31 | 0.027973 | 0.030356 | 0.356795 |
| rs56335308 | 8 | 17419461 | A | 0.117482 | 0.012633 | 1.40E-20 | 0.00018 | 0.035959 | 0.996014 |
| rs58673065 | 7 | 1885600 | G | 0.029884 | 0.004902 | 1.10E-09 | 0.004742 | 0.02115 | 0.822578 |
| rs62182473 | 2 | 1.92E+08 | T | -0.07072 | 0.00469 | 2.20E-51 | 0.026298 | 0.018308 | 0.15087 |
| rs7078003 | 10 | 99359412 | T | 0.104082 | 0.0054 | 8.80E-83 | 0.062744 | 0.039931 | 0.116108 |
| rs7147721 | 14 | 75186010 | G | 0.026091 | 0.004151 | 3.30E-10 | -0.00555 | 0.018282 | 0.761286 |
| rs738408 | 22 | 44324730 | T | 0.03237 | 0.005008 | 1.00E-10 | -0.01387 | 0.018055 | 0.442312 |
| rs7925445 | 11 | 18398958 | G | 0.028546 | 0.004165 | 7.20E-12 | 0.007673 | 0.018648 | 0.680725 |
| rs7966322 | 12 | 1.21E+08 | T | -0.02443 | 0.00445 | 4.00E-08 | 0.043642 | 0.030242 | 0.148994 |
| rs838737 | 2 | 2.34E+08 | A | -0.02346 | 0.004162 | 1.70E-08 | -0.01872 | 0.019325 | 0.332786 |
| rs904538 | 17 | 25591429 | A | 0.027066 | 0.004139 | 6.20E-11 | -0.0077 | 0.02135 | 0.718484 |
| rs9482770 | 6 | 1.27E+08 | C | -0.03234 | 0.004166 | 8.40E-15 | -0.018 | 0.018225 | 0.323372 |

SNP single nucleotide polymorphism; Chr chromosome; EA effect allele; SE standard error

Table S5. Characteristic of the Glutamine-related genetic variants and their effects on Liver cell carcinoma (28 SNPs)

| SNP | Chr | Position | EA | SNPs-Glutamine | | | SNPs- Liver cell carcinoma | | |
| --- | --- | --- | --- | --- | --- | --- | --- | --- | --- |
|  |  |  |  | β | SE | P value | β | SE | P value |
| rs10811663 | 9 | 22139220 | A | -0.04815 | 0.008046 | 2.20E-09 | -0.00013599 | 9.58E-05 | 0.16 |
| rs10849008 | 12 | 4301876 | C | 0.030089 | 0.004264 | 1.70E-12 | 1.62E-05 | 5.08E-05 | 0.75 |
| rs112081903 | 16 | 70014459 | C | -0.02534 | 0.004514 | 2.00E-08 | -4.27E-05 | 5.39E-05 | 0.43 |
| rs11386117 | 10 | 1.15E+08 | CA | -0.02421 | 0.004133 | 4.70E-09 | -2.93E-05 | 4.94E-05 | 0.55 |
| rs11422720 | 6 | 1.32E+08 | GC | -0.03616 | 0.00498 | 3.80E-13 | -3.68E-05 | 5.93E-05 | 0.54 |
| rs11993225 | 8 | 1.34E+08 | C | 0.025967 | 0.004637 | 2.10E-08 | -2.35E-05 | 5.54E-05 | 0.67 |
| rs1260326 | 2 | 27730940 | C | 0.078942 | 0.004218 | 3.60E-78 | -0.00012256 | 5.03E-05 | 0.015 |
| rs1274961 | 3 | 39191335 | C | -0.02967 | 0.005001 | 3.00E-09 | -7.38E-05 | 5.98E-05 | 0.22 |
| rs13094915 | 3 | 52507719 | C | 0.025648 | 0.004312 | 2.70E-09 | 2.43E-06 | 5.13E-05 | 0.96 |
| rs1323320 | 6 | 56287985 | A | -0.02588 | 0.004687 | 3.40E-08 | -2.88E-05 | 5.60E-05 | 0.61 |
| rs2039098 | 20 | 56115153 | T | -0.02416 | 0.004195 | 8.40E-09 | -3.09E-05 | 4.95E-05 | 0.53 |
| rs2168101 | 11 | 8255408 | A | -0.06586 | 0.00461 | 2.70E-46 | 7.17E-05 | 5.48E-05 | 0.19 |
| rs2657879 | 12 | 56865338 | G | -0.25113 | 0.005326 | 1.00E-200 | 6.73E-05 | 6.36E-05 | 0.29 |
| rs28362590 | 5 | 1.77E+08 | T | -0.02621 | 0.004798 | 4.70E-08 | -1.44E-05 | 5.71E-05 | 0.8 |
| rs35007880 | 14 | 1.01E+08 | T | 0.033105 | 0.004167 | 2.00E-15 | 3.53E-05 | 4.97E-05 | 0.48 |
| rs35213094 | 12 | 59945417 | A | 0.065283 | 0.005919 | 2.80E-28 | 1.58E-05 | 7.05E-05 | 0.82 |
| rs35261542 | 6 | 20675792 | A | 0.030213 | 0.004705 | 1.40E-10 | -9.88E-06 | 5.60E-05 | 0.86 |
| rs3812316 | 7 | 73020337 | G | 0.070825 | 0.006143 | 9.30E-31 | 3.51E-05 | 7.37E-05 | 0.630001 |
| rs4237150 | 9 | 4290541 | C | 0.033008 | 0.004206 | 4.20E-15 | 0.000121407 | 5.02E-05 | 0.016 |
| rs4365129 | 12 | 47237515 | T | -0.02657 | 0.004317 | 7.50E-10 | 5.55E-05 | 5.15E-05 | 0.28 |
| rs58673065 | 7 | 1885600 | G | 0.029884 | 0.004902 | 1.10E-09 | -1.58E-05 | 5.86E-05 | 0.79 |
| rs62182473 | 2 | 1.92E+08 | T | -0.07072 | 0.00469 | 2.20E-51 | 2.27E-05 | 5.59E-05 | 0.68 |
| rs7078003 | 10 | 99359412 | T | 0.104082 | 0.0054 | 8.80E-83 | 2.76E-05 | 6.43E-05 | 0.67 |
| rs7147721 | 14 | 75232306 | G | 0.026091 | 0.004151 | 3.30E-10 | 2.43E-05 | 4.95E-05 | 0.62 |
| rs7925445 | 11 | 18398958 | G | 0.028546 | 0.004165 | 7.20E-12 | -3.58E-05 | 4.97E-05 | 0.47 |
| rs838737 | 2 | 2.34E+08 | A | -0.02346 | 0.004162 | 1.70E-08 | 1.10E-05 | 4.96E-05 | 0.83 |
| rs904538 | 17 | 25595818 | A | 0.027066 | 0.004139 | 6.20E-11 | 1.03E-05 | 4.95E-05 | 0.84 |
| rs9482770 | 6 | 1.27E+08 | C | -0.03234 | 0.004166 | 8.40E-15 | 1.53E-05 | 4.96E-05 | 0.760001 |

SNP single nucleotide polymorphism; Chr chromosome; EA effect allele; SE standard error

Table S6. Characteristic of the Glutamine-related genetic variants and their effects on Hepatic bile duct cancer (32 SNPs)

| SNP | Chr | Position | EA | SNPs-Glutamine | | | SNPs- Hepatic bile duct cancer | | |
| --- | --- | --- | --- | --- | --- | --- | --- | --- | --- |
|  |  |  |  | β | SE | P value | β | SE | P value |
| rs10811663 | 9 | 22139220 | A | -0.048147 | 0.00804615 | 2.20E-09 | 0.0833197 | 0.0936563 | 0.373663 |
| rs10849008 | 12 | 4301876 | C | 0.0300893 | 0.00426401 | 1.70E-12 | -0.0149727 | 0.0715111 | 0.834155 |
| rs112081903 | 16 | 70014459 | C | -0.0253394 | 0.00451361 | 2.00E-08 | 0.0354774 | 0.0773417 | 0.646443 |
| rs113674212 | 12 | 47198856 | A | 0.134105 | 0.00819183 | 3.10E-60 | -0.0781036 | 0.152234 | 0.607917 |
| rs11386117 | 10 | 114772253 | CA | -0.0242117 | 0.00413313 | 4.70E-09 | -0.178634 | 0.135452 | 0.187237 |
| rs11422720 | 6 | 131877966 | GC | -0.0361623 | 0.00498015 | 3.80E-13 | -0.00100227 | 0.0735487 | 0.989127 |
| rs11993225 | 8 | 134335352 | C | 0.0259672 | 0.00463689 | 2.10E-08 | 0.0554379 | 0.1255 | 0.658681 |
| rs1260326 | 2 | 27730940 | C | 0.0789422 | 0.00421761 | 3.60E-78 | 0.0489325 | 0.0697427 | 0.48292 |
| rs1274961 | 3 | 39191335 | C | -0.0296721 | 0.00500127 | 3.00E-09 | -0.683069 | 0.919102 | 0.457365 |
| rs1323320 | 6 | 56287985 | A | -0.0258759 | 0.00468672 | 3.40E-08 | -0.0189154 | 0.0700311 | 0.787084 |
| rs1750768 | 10 | 22965448 | T | 0.0565226 | 0.0103452 | 4.70E-08 | 0.511823 | 0.679686 | 0.451433 |
| rs1998848 | 14 | 21492229 | A | 0.166954 | 0.0209281 | 1.50E-15 | -0.0303414 | 0.101437 | 0.764851 |
| rs2039098 | 20 | 56115153 | T | -0.0241636 | 0.00419511 | 8.40E-09 | -0.0170064 | 0.0721918 | 0.813765 |
| rs2168101 | 11 | 8255408 | A | -0.0658561 | 0.00460998 | 2.70E-46 | 0.0528784 | 0.0854612 | 0.536087 |
| rs2657879 | 12 | 56865338 | G | -0.251133 | 0.0053259 | 1.00E-200 | 0.179461 | 0.1134 | 0.113525 |
| rs28362590 | 5 | 176731452 | T | -0.0262054 | 0.00479807 | 4.70E-08 | -0.0316803 | 0.0693466 | 0.647787 |
| rs35007880 | 14 | 100821994 | T | 0.0331051 | 0.00416726 | 2.00E-15 | -0.0162274 | 0.0935789 | 0.86233 |
| rs35213094 | 12 | 59964919 | A | 0.0652828 | 0.00591895 | 2.80E-28 | 0.148707 | 1.09405 | 0.891882 |
| rs35261542 | 6 | 20675792 | A | 0.0302132 | 0.00470514 | 1.40E-10 | -0.00801359 | 0.0702568 | 0.909189 |
| rs3812316 | 7 | 73020337 | G | 0.0708251 | 0.00614256 | 9.30E-31 | -0.032205 | 0.116279 | 0.78181 |
| rs4365129 | 12 | 47229840 | T | -0.0265688 | 0.00431716 | 7.50E-10 | -0.084594 | 0.0720891 | 0.24061 |
| rs56335308 | 8 | 17419461 | A | 0.117482 | 0.0126332 | 1.40E-20 | 0.0551973 | 0.13834 | 0.689895 |
| rs58673065 | 7 | 1885600 | G | 0.0298844 | 0.00490242 | 1.10E-09 | -0.0854007 | 0.0810015 | 0.291741 |
| rs62182473 | 2 | 191740996 | T | -0.0707191 | 0.00468972 | 2.20E-51 | -0.0126268 | 0.0703013 | 0.857459 |
| rs7078003 | 10 | 99359412 | T | 0.104082 | 0.00539996 | 8.80E-83 | 0.0114218 | 0.153266 | 0.940594 |
| rs7147721 | 14 | 75232306 | G | 0.026091 | 0.00415066 | 3.30E-10 | -0.0923578 | 0.0694097 | 0.183315 |
| rs738408 | 22 | 44324730 | T | 0.0323695 | 0.00500826 | 1.00E-10 | 0.09274 | 0.0695699 | 0.182516 |
| rs7925445 | 11 | 18398958 | G | 0.0285458 | 0.00416485 | 7.20E-12 | 0.0121903 | 0.0714142 | 0.86446 |
| rs7966322 | 12 | 121401846 | T | -0.0244282 | 0.00445027 | 4.00E-08 | -0.122917 | 0.113955 | 0.280746 |
| rs838737 | 2 | 234325052 | A | -0.0234551 | 0.00416218 | 1.70E-08 | -0.103198 | 0.0730634 | 0.157819 |
| rs904538 | 17 | 25591429 | A | 0.0270655 | 0.00413851 | 6.20E-11 | -0.0863903 | 0.0821804 | 0.293155 |
| rs9482770 | 6 | 127443092 | C | -0.0323379 | 0.00416649 | 8.40E-15 | -0.00927474 | 0.069743 | 0.894206 |

SNP single nucleotide polymorphism; Chr chromosome; EA effect allele; SE standard error

Table S7. Characteristic of the Glutamine-related genetic variants and their effects on Pancreatic cancer (41 SNPs)

| SNP | Chr | Position | EA | SNPs-Glutamine | | | SNPs- Pancreatic cancer | | |
| --- | --- | --- | --- | --- | --- | --- | --- | --- | --- |
|  |  |  |  | β | SE | P value | β | SE | P value |
| rs10811663 | 9 | 22139220 | A | -0.04815 | 0.008046 | 2.20E-09 | -0.0405 | 0.0565 | 0.4737 |
| rs10849008 | 12 | 4301876 | C | 0.030089 | 0.004264 | 1.70E-12 | 0.0203 | 0.0403 | 0.614601 |
| rs112081903 | 16 | 70014459 | C | -0.02534 | 0.004514 | 2.00E-08 | 0.008 | 0.0382 | 0.8334 |
| rs113674212 | 12 | 47198856 | A | 0.134105 | 0.008192 | 3.10E-60 | 0.0296 | 0.0697 | 0.6715 |
| rs11386117 | 10 | 1.15E+08 | CA | -0.02421 | 0.004133 | 4.70E-09 | 0.0113 | 0.0392 | 0.7739 |
| rs11422720 | 6 | 1.32E+08 | GC | -0.03616 | 0.00498 | 3.80E-13 | -0.0092 | 0.0392 | 0.815 |
| rs117001881 | 12 | 57304853 | A | -0.12046 | 0.021557 | 2.30E-08 | -0.3326 | 0.1933 | 0.085349 |
| rs11993225 | 8 | 1.34E+08 | C | 0.025967 | 0.004637 | 2.10E-08 | -0.0216 | 0.043 | 0.615601 |
| rs1260326 | 2 | 27730940 | C | 0.078942 | 0.004218 | 3.60E-78 | 0.0532 | 0.0354 | 0.1326 |
| rs1274961 | 3 | 39191335 | C | -0.02967 | 0.005001 | 3.00E-09 | 0.0234 | 0.0485 | 0.6291 |
| rs13094915 | 3 | 52507719 | C | 0.025648 | 0.004312 | 2.70E-09 | 0.0452 | 0.0435 | 0.2992 |
| rs1323320 | 6 | 56287985 | A | -0.02588 | 0.004687 | 3.40E-08 | -0.0492 | 0.0378 | 0.1928 |
| rs13396296 | 2 | 2.11E+08 | C | -0.02419 | 0.004342 | 2.50E-08 | 0.0061 | 0.0518 | 0.9059 |
| rs142525555 | 12 | 57232374 | AT | -0.06146 | 0.009776 | 3.20E-10 | -0.1932 | 0.1054 | 0.06677 |
| rs17096421 | 10 | 88820592 | T | -0.05138 | 0.00907 | 1.50E-08 | -0.0251 | 0.0945 | 0.7903 |
| rs1750768 | 10 | 22926227 | T | 0.056523 | 0.010345 | 4.70E-08 | 0.0624 | 0.106 | 0.5561 |
| rs1998848 | 14 | 21492229 | A | 0.166954 | 0.020928 | 1.50E-15 | 0.1314 | 0.0767 | 0.086581 |
| rs2039098 | 20 | 56112882 | T | -0.02416 | 0.004195 | 8.40E-09 | 0.0041 | 0.0503 | 0.9355 |
| rs2168101 | 11 | 8255408 | A | -0.06586 | 0.00461 | 2.70E-46 | -0.0229 | 0.039 | 0.5563 |
| rs2657879 | 12 | 56865338 | G | -0.25113 | 0.005326 | 1.00E-200 | 0.0432 | 0.0474 | 0.3621 |
| rs28362590 | 5 | 1.77E+08 | T | -0.02621 | 0.004798 | 4.70E-08 | 0.015 | 0.0379 | 0.691 |
| rs28929474 | 14 | 94844947 | T | 0.090546 | 0.014775 | 8.90E-10 | 0.1285 | 0.1469 | 0.3816 |
| rs35007880 | 14 | 1.01E+08 | T | 0.033105 | 0.004167 | 2.00E-15 | 0.0299 | 0.0416 | 0.4711 |
| rs35213094 | 12 | 59945417 | A | 0.065283 | 0.005919 | 2.80E-28 | -0.0569 | 0.0707 | 0.4203 |
| rs35261542 | 6 | 20675792 | A | 0.030213 | 0.004705 | 1.40E-10 | 0.0286 | 0.0374 | 0.4441 |
| rs3812316 | 7 | 73020337 | G | 0.070825 | 0.006143 | 9.30E-31 | 0.0788 | 0.0531 | 0.1379 |
| rs4237150 | 9 | 4290085 | C | 0.033008 | 0.004206 | 4.20E-15 | -0.0044 | 0.0351 | 0.8995 |
| rs4365129 | 12 | 47229840 | T | -0.02657 | 0.004317 | 7.50E-10 | -0.0242 | 0.0364 | 0.5059 |
| rs56335308 | 8 | 17419461 | A | 0.117482 | 0.012633 | 1.40E-20 | 0.0849 | 0.094 | 0.3666 |
| rs58673065 | 7 | 1885600 | G | 0.029884 | 0.004902 | 1.10E-09 | -0.0302 | 0.0403 | 0.4539 |
| rs62182473 | 2 | 1.92E+08 | T | -0.07072 | 0.00469 | 2.20E-51 | 0.0096 | 0.0381 | 0.8013 |
| rs7078003 | 10 | 99359412 | T | 0.104082 | 0.0054 | 8.80E-83 | 0.0068 | 0.0498 | 0.8912 |
| rs7147721 | 14 | 75186010 | G | 0.026091 | 0.004151 | 3.30E-10 | -0.0768 | 0.0496 | 0.1216 |
| rs738408 | 22 | 44324730 | T | 0.03237 | 0.005008 | 1.00E-10 | -0.0188 | 0.0393 | 0.6329 |
| rs78431863 | 11 | 74109553 | T | 0.079422 | 0.010427 | 2.60E-14 | -0.1792 | 0.1257 | 0.1542 |
| rs7925445 | 11 | 18398958 | G | 0.028546 | 0.004165 | 7.20E-12 | 0.0075 | 0.0397 | 0.8509 |
| rs7966322 | 12 | 1.21E+08 | T | -0.02443 | 0.00445 | 4.00E-08 | 0.0439 | 0.1052 | 0.676401 |
| rs79687284 | 1 | 2.14E+08 | C | 0.155571 | 0.011304 | 4.30E-43 | 0.1529 | 0.1222 | 0.2108 |
| rs838737 | 2 | 2.34E+08 | A | -0.02346 | 0.004162 | 1.70E-08 | -0.0198 | 0.0353 | 0.5745 |
| rs904538 | 17 | 25591429 | A | 0.027066 | 0.004139 | 6.20E-11 | -0.0665 | 0.0363 | 0.067281 |
| rs9482770 | 6 | 1.27E+08 | C | -0.03234 | 0.004166 | 8.40E-15 | 0.0581 | 0.0348 | 0.09503 |

SNP single nucleotide polymorphism; Chr chromosome; EA effect allele; SE standard error
