# Supplementary figures and images for: Causal effects of circulating glutamine on colitis, IBD, and digestive system cancers: a Mendelian randomisation study
Source: J Cancer. 2024 May 20;15(12):3738–49. doi: 10.7150/jca.96085 (PMC11190753; doi:10.7150/jca.96085)

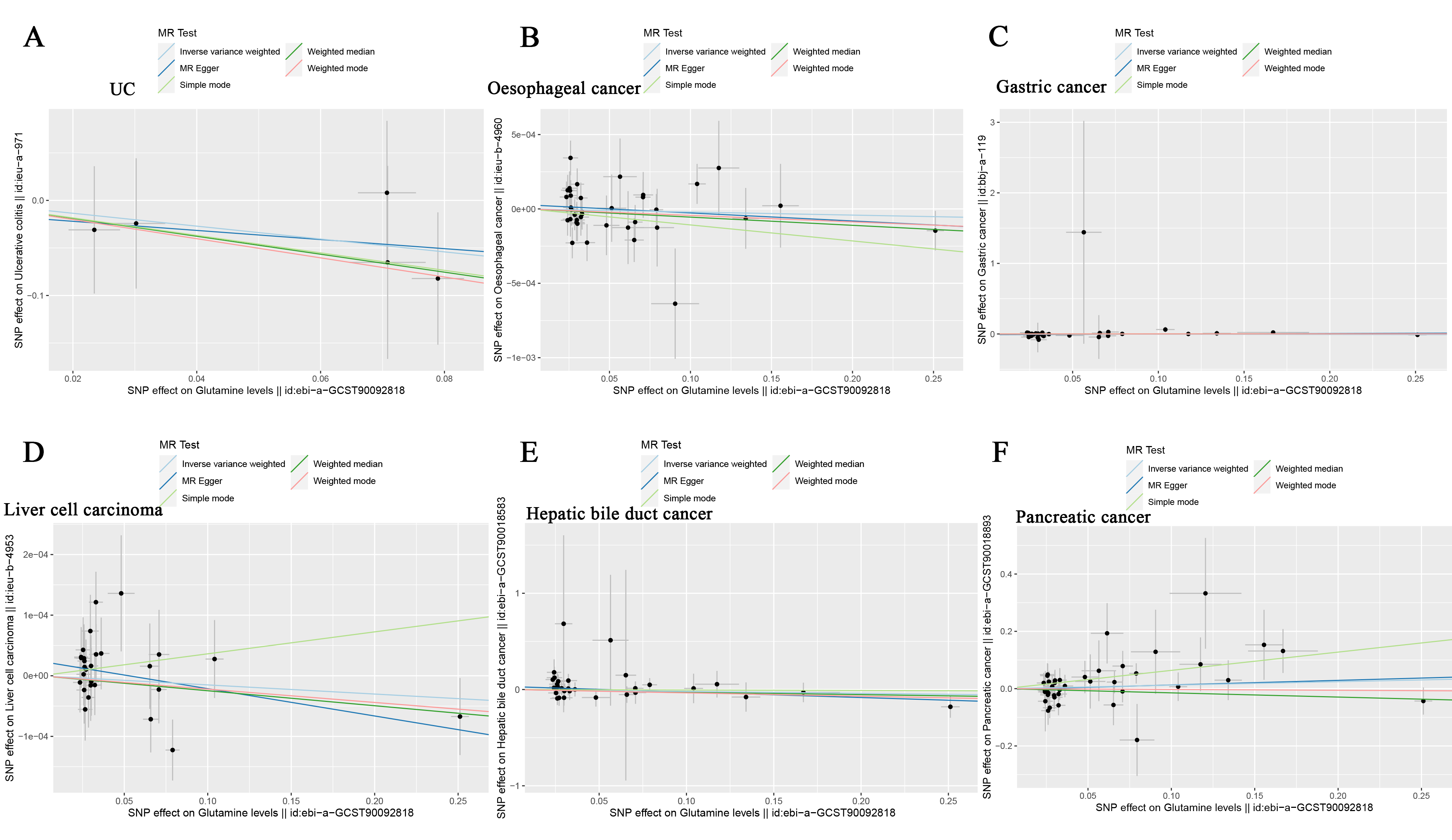

Supplement: Supplementary file 1 — Supplementary figures and tables. [file jcav15p3738s1.zip › Supplementary Figure 1.tif]

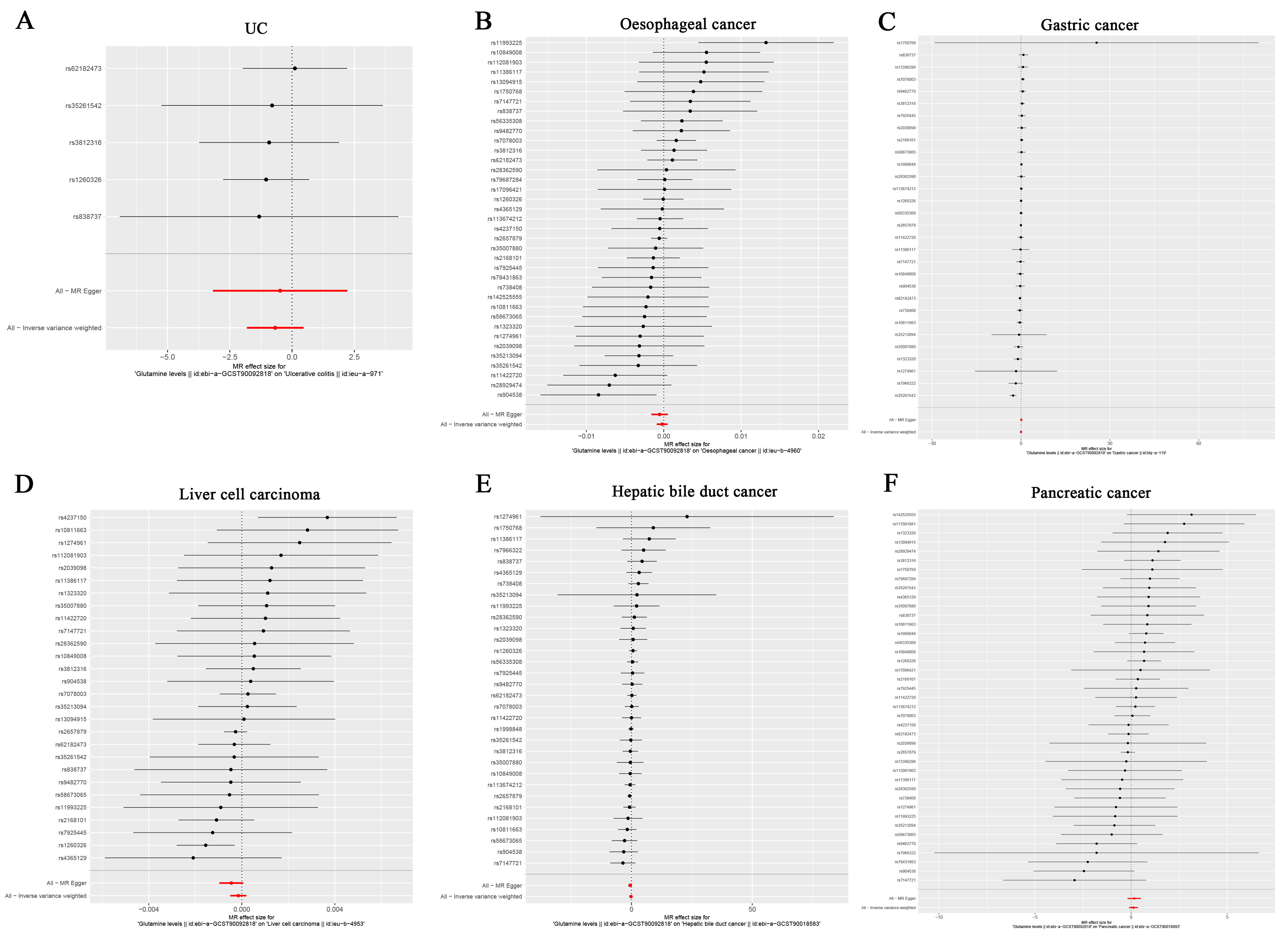

Supplement: Supplementary file 1 — Supplementary figures and tables. [file jcav15p3738s1.zip › Supplementary Figure 2.tif]

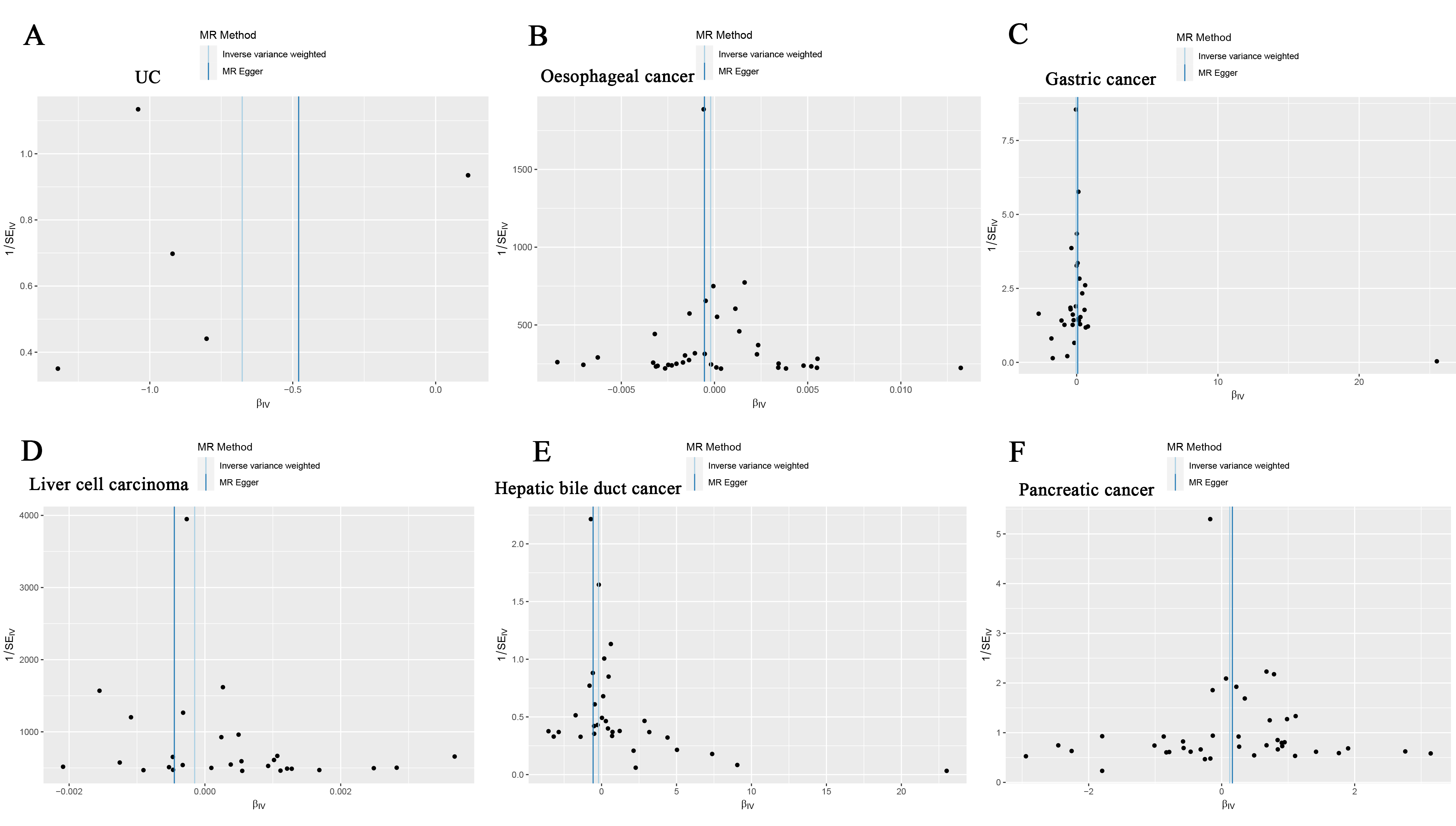

Supplement: Supplementary file 1 — Supplementary figures and tables. [file jcav15p3738s1.zip › Supplementary Figure 3.tif]

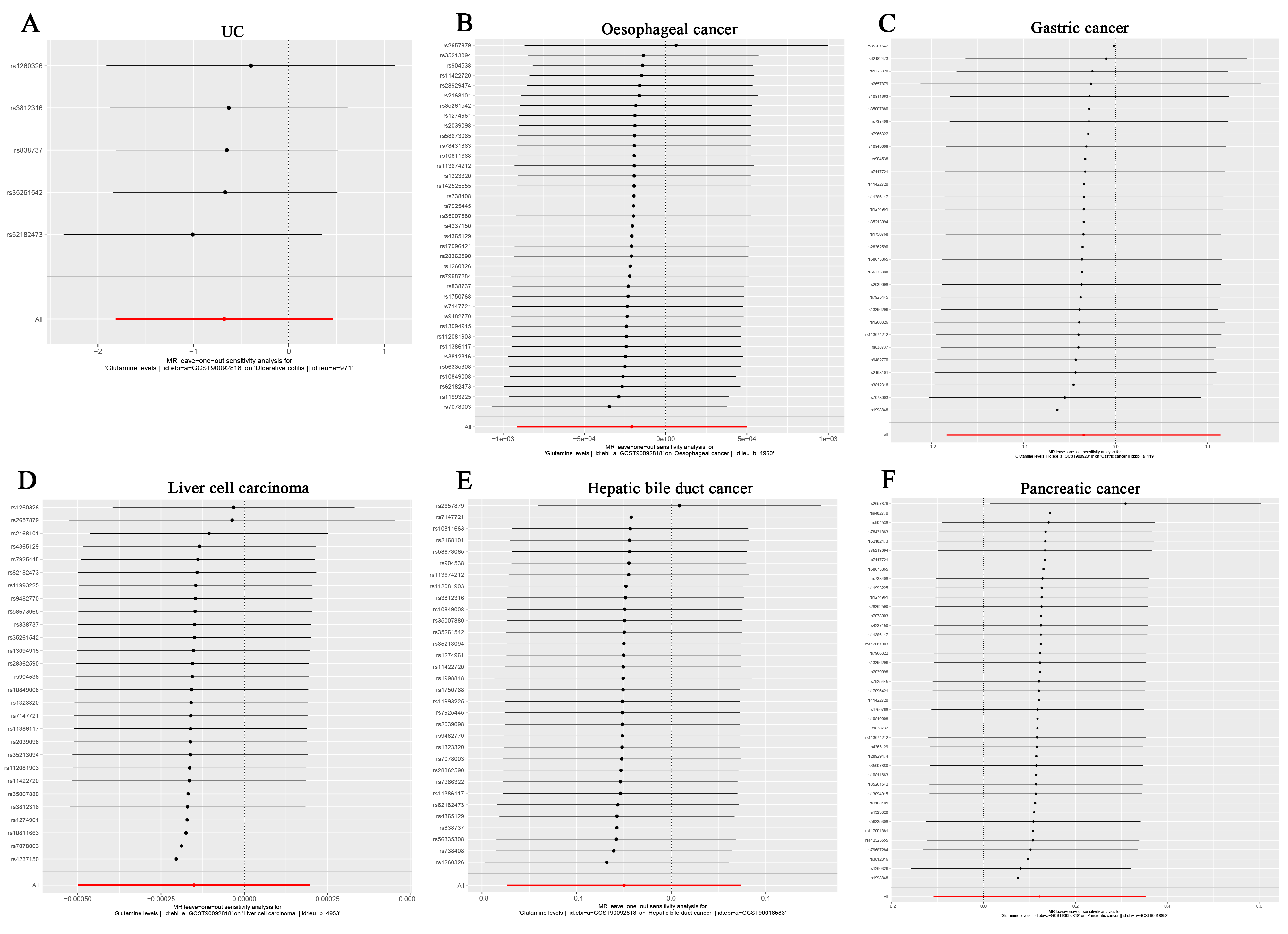

Supplement: Supplementary file 1 — Supplementary figures and tables. [file jcav15p3738s1.zip › Supplementary Figure 4.tif]
